# Transcriptome profiling of a spirodiclofen susceptible and resistant strain of the European red mite *Panonychus ulmi* using strand-specific RNA-seq

**DOI:** 10.1186/s12864-015-2157-1

**Published:** 2015-11-18

**Authors:** Sabina Bajda, Wannes Dermauw, Robert Greenhalgh, Ralf Nauen, Luc Tirry, Richard M. Clark, Thomas Van Leeuwen

**Affiliations:** Institute for Biodiversity and Ecosystem Dynamics, University of Amsterdam, P.O. Box 9424, 1090 GE Amsterdam, The Netherlands; Laboratory of Agrozoology, Department of Crop Protection, Faculty of Bioscience Engineering, Ghent University, Coupure Links 653, B-9000 Ghent, Belgium; Department of Biology, University of Utah, Salt Lake City, 257 South 1400 East, UT 84112 USA; Bayer CropScience AG, Research Pest Control, Alfred Nobel Str. 50, D-40789 Monheim, Germany; Center for Cell and Genome Science, University of Utah, Salt Lake City, 257 South 1400 East, UT 84112 USA

**Keywords:** Cyclic keto-enols, Tetranychidae, Target-site mutation, biRNA virus, Spirotetramat, Intradiol ring-cleavage dioxygenases, UDP-glycosyltransferases, Cytochrome P450 mono-oxygenases, Carboxyl/cholinesterases, Gluthathione-S-transferases

## Abstract

**Background:**

The European red mite, *Panonychus ulmi*, is among the most important mite pests in fruit orchards, where it is controlled primarily by acaricide application. However, the species rapidly develops pesticide resistance, and the elucidation of resistance mechanisms for *P. ulmi* has not kept pace with insects or with the closely related spider mite *Tetranychus urticae*. The main reason for this lack of knowledge has been the absence of genomic resources needed to investigate the molecular biology of resistance mechanisms.

**Results:**

Here, we provide a comprehensive strand-specific RNA-seq based transcriptome resource for *P. ulmi* derived from strains susceptible and resistant to the widely used acaricide spirodiclofen. From a *de novo* assembly of the *P. ulmi* transcriptome, we manually annotated detoxification enzyme families, target-sites of commonly used acaricides, and horizontally transferred genes implicated in plant-mite interactions and pesticide resistance. In a comparative analysis that incorporated sequences available for *Panonychus citri*, *T. urticae*, and insects, we identified radiations for detoxification gene families following the divergence of *Panonychus* and *Tetranychus* genera*.* Finally, we used the replicated RNA-seq data from the spirodiclofen susceptible and resistant strains to describe gene expression changes associated with resistance. A cytochrome P450 monooxygenase, as well as multiple carboxylcholinesterases, were differentially expressed between the susceptible and resistant strains, and provide a molecular entry point for understanding resistance to spirodiclofen, widely used to control *P. ulmi* populations.

**Conclusions:**

The new genomic resources and data that we present in this study for *P. ulmi* will substantially facilitate molecular studies of underlying mechanisms involved in acaricide resistance.

**Electronic supplementary material:**

The online version of this article (doi:10.1186/s12864-015-2157-1) contains supplementary material, which is available to authorized users.

## Background

The European red mite, *Panonychus ulmi,* is a member of the family Tetranychidae (Arthropoda: Chelicerata: Arachnida: Acari). Tetranychidae, or spider mites, use their stylet- like chelicerae to puncture the leaf mesophyll-cells to suck out cell contents. This results in chlorotic spots and necrosis and ultimately, leaf abscission [1]. As a result, among mite pests, spider mites are economically important with 80 % of the acaricide market used for their control [2]. Nonetheless, a major problem associated with spider mite control is the rapid development of acaricide resistance. Factors accelerating resistance evolution include not only the frequent use of acaricides, but also high fecundity rates, arrhenotokous reproduction (on haploid males selection is highly effective) and a short life cycle [3]. As a result, the two-spotted spider mite (*Tetranychus urticae*) and *P. ulmi* have developed resistance to nearly all acaricide classes [4] and rank among the top 10 of most resistant arthropod species based on the number of unique active ingredients for which resistance has been reported [2].

The acaricides spirodiclofen and spiromesifen belong, together with the insecticide spirotetramat, to the family of spirocyclic tetronic acids (keto-enols) and interrupt lipid biosynthesis by direct inhibition of acetyl coenzyme A carboxylase (ACCase) [5, 6]. Introduced relatively recently to the market [7], spirodiclofen has been shown to be an effective tool in resistance management of tetranychid mites. It shows good to excellent residual control [8, 9] and is effective against spider mites resistant to different acaricide classes [10–16]. Although high levels of spirodiclofen resistance have not yet been reported in field populations, artificial selection studies have shown that spider mites have the potential to develop resistance to spirodiclofen comparatively rapid in the laboratory [17, 18]. Selection for resistance was undertaken with *T. urticae*, *P. ulmi* and *P. citri* and resistance in all species was synergized by piperonylbutoxide (PBO), suggesting the involvement of cytochrome P450s [17–19]. Strikingly, for all three tetranychid species spirodiclofen resistance was age dependent. The concentration of spirodiclofen causing 50 % lethality (LC_50_) in the resistant strains was much lower in eggs then in mobile stages, a finding Demaeght et al. [20] suggested could be due to lower expression of detoxification genes in eggs.

In 2011, the high quality genome sequence of the spider mite *Tetranychus urticae* became available [21], and afforded new resources and tools to investigate the molecular mechanisms underlying acaricide resistance [20, 22–25]. For example, although biochemical studies had shown that elevated P450 and esterase levels were associated with resistance to spirodiclofen in *T. urticae* (see above), studies with a genome-wide gene expression microarray allowed the identification of the genes coding for the detoxification enzymes associated with these activities (*CYP392E10* and *TuCCE-04*) [20]. It is clear that our understanding of resistance to acaricides in phytophagous mites improved with the advent of the *T. urticae* genome, however, such genomic resources are scarce for other key tetranychid mites, particularly *P. ulmi*. With only 49 nucleotide sequences available in the NCBI public database (accessed May 2015), *P. ulmi* resistance research at the molecular level has hardly been possible.

For organisms without a sequenced genome, *de novo* transcriptome assemblies provide an important starting point for genomic analyses, and recent advances in high-throughput transcript sequencing have greatly contributed to our understanding of gene expression and structure [26–29]. This is especially true in agricultural pest management, where the majority of pest species do not have a reference genome and where the elucidation of resistance mechanisms of insecticides is vital [30–34].

In this study we used the Illumina HiSeq2000 technology to generate deep paired-end, strand-specific RNA-seq reads for two strains of *P. ulmi*. The first strain (HS) is susceptible to most currently used acaricides, while the second strain (PSR-TK) was field collected and shown to be resistant to a number of acaricides, including hexythiazox and clofentezine. The strain also showed decreased susceptibility to spirodiclofen, and was further selected in the laboratory until high levels of spirodiclofen resistance (Resistance ratio of 7000) were reached [17]. Paired-end RNAseq reads were used to assemble a *P. ulmi* transcriptome, and with the same assembly methodology we constructed two *P. citri* transcriptomes based on previously published RNA-seq data available as single end reads [33, 34]. We then performed a phylogenetic classification of *Panonychus* gene families involved in detoxification, such as cytochrome P450 monooxygenases (CYPs), carboxylcholinesterases (CCEs), glutathione-S-transferases (GSTs), UDP-glycosyl transferases (UGTs) and ABC transporters (ABCs), and searched for horizontally transferred genes that were previously uncovered in the genome sequence of *T. urti*cae [21, 35–39]. Moreover, a differential expression analysis between the acaricide susceptible and resistant *P. ulmi* strain allowed us to identify genes encoding proteins that are candidates for involvement in acaricide resistance. In addition, we identified and compared *P. ulmi* expressed sequences for known acaricide target sites. The assembled annotated transcriptome of *P. ulmi* provides an important resource that should substantially facilitate molecular studies in this species, including the elucidation of detoxification mechanisms of xenobiotics.

## Results and discussion

### *De novo* sequence assembly

We performed high-throughput Illumina sequencing of RNA extracted from adult females from acaricide susceptible (HS) and resistant *P. ulmi* strains (PSR-TK). With the Illumina HiSeq platform we generated 66,308,047 and 95,412,934 strand-specific paired-end reads for the HS and PSR-TK *P. ulmi* strains, respectively. Two approaches were used to assemble the *P. ulmi* transcriptome (see Methods for details). The assembly created with CLC Genomics Workbench (CLC) contains 27,777 unique *P. ulmi* contigs longer than 200 bp, totalling 27.6 Mb of sequence with an overall GC content of 33.6 %. The average contig size was 993 bp and the N50 was 2087 bp. The Velvet/Oases assembly, on the other hand, consists of 30,044 transcripts totalling 18.6 Mb of sequence with a GC content of 34.5 % and an average contig size/N50 of 619 bp/871 bp. Mapping all RNA-seq reads against both transcriptomes (CLC or Velvet/Oases) revealed that the overall mapping success rate, as a measure for assembly quality, was significantly lower for the Velvet/Oases assembly compared to the CLC assembly (see Additional file 1), with an average overall alignment rate of 91.7 % and 73.4 % for the CLC and Velvet/Oases assembly, respectively. Hence, unless otherwise stated, the CLC assembly was used for all further analyses described in this study. All raw reads and the CLC assembly were submitted to NCBI under BioProject PRJNA271858.

As the transcriptome assemblies of two previously published *P. citri* transcriptomes [33, 34] were not publicly available, and to generate assemblies with similar methodology, we assembled these transcriptomes in a similar way (CLC) as for *P. ulmi* (Additional files 2 and 3). *P. citri* assembly statistics were in line with those of *P. ulmi,* with 25,529 contigs and an average contig size/N50 of 708 bp/1057 bp for the Liu et al. [33] *P. citri* transcriptome and 32,362 contigs and an average contig size/N50 of 646 bp/969 bp for the Niu et al. [34] *P. citri* transcriptome.

### Homology searches, species distribution and Gene Ontology (GO) analysis

A total of 9190 sequences (33.1 % of all contigs) had a BLAST hit in the NCBI non-redundant (nr) database, with 9109 sequences having a BLASTx hit against the nr protein database and 81 sequences having a BLASTn hit against the nr nucleotide database. As *T. urticae* gene annotations have not yet been uploaded to the NCBI database, we also performed a local BLASTx search against the *T. urticae* proteome. We found that 11,250 *P. ulmi* contigs showed a BLASTx hit with the predicted *T. urticae* proteome, including 2483 that had no BLASTx-hit in the NCBI nr database. In total, 11,673 *P. ulmi* contigs (42,0 % of all contigs) had a BLAST-hit in at least one analysis (Additional file 4). Although 42.0 % of contigs with a BLAST-hit may appear to be a relatively small number, we found that this value does not significantly differ from other *de novo* transcriptome studies with non-model arthropod species (e.g. in [40, 41]). In the Niu et al. transcriptome study [34] of the related *P. citri* the percentage of contigs was also of similar magnitude (50.7 %). Such a low percentage can be caused by multiple reasons. The majority of *de novo* transcriptomes contains a considerable amount of short contigs (<240 bp), containing incomplete (<80 AA) ORFs that in many cases will not BLAST at a given E-value threshold. On the other hand some contigs may be non-coding RNAs which do not BLAST with the non-redundant protein/nucleotide database (or just UTR fragments of otherwise coding genes).

Out of the contigs having a BLAST-hit, 94.1 % (10,989 contigs) of the top BLAST hits belonged to metazoan species, 2.9 % to Bacteria (342 contigs), 1.6 % (181 contigs) to Plants, 0.6 % (75 contigs) to Fungi and 0.7 % (86 contigs) to other organisms. The highest homologies for the majority of the metazoan BLAST hits belonged to the Chelicerata (55.7 %, 6498 contigs) and Arthropoda (20.6 %, 2404 contigs) (Additional file 5). A detailed analysis of all chelicerate BLAST hits revealed a high number of top BLAST hits with *T. urticae* (21.9 %, 2553 contigs), followed by the social velvet spider *Stegodyphus mimosarum* (17.8 %, 2080 contigs), the tick *Ixodes scapularis* (8.5 %, 987 contigs) and the predatory mite *Metaseiulus occidentalis* (4.7 %, 545 contigs).

**Fig. 1:**
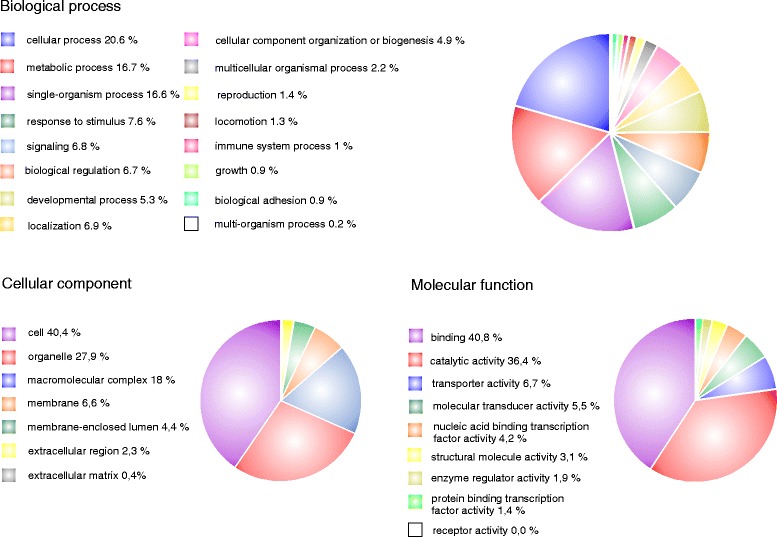
Gene ontology annotation and classification (level 2) of the *P. ulmi* transcriptome. Results are summarized into three main categories: molecular function, biological process and cellular component. In total GO terms were assigned to 5833 *P. ulmi* contigs

We performed a Gene Ontology (GO) analysis of the *P. ulmi* assembled sequences after excluding contigs (227) that were considered as foreign contamination and consisted mainly of sequences belonging to *Wolbachia* sp. (111 contigs) and the host plant (*Prunus* sp.) of the *P. ulmi* HS and PSR-TK strains (79 contigs) (see Methods). GO terms were assigned to 5833 unique contigs (21.2 % of 27,550 contigs) and in most cases multiple GO terms were assigned to the same *P. ulmi* contig. We found that 17,161 (54.8 %), 6623 (21.2 %) and 7534 (24.1 %) GO terms emerged for biological process, cellular component and molecular function categories, respectively. Those were further categorized into 16 biological process, 9 molecular function and 7 cellular component sub-categories (Fig. 1). Among the 16 sub-categories of biological process, the cellular process occupied the highest number (3535, 20.6 %), followed by the metabolic process (2858, 16.7 %), single organism process (2853, 16.6 %) and response to stimulus (1308, 7.6 %) (Fig. 1). The major sub-categories of the molecular function category included binding (2705, 40.8 %), followed by catalytic activity (2409, 36.4 %) and transporter activity (445, 6.7 %) (Fig. 1). In the cellular component domain, the majority of the GO terms were shown to be specific for the cell (3047, 40.4 %), followed by organelle (2101, 27.9 %) and macromolecular complex (1355, 18,0 %) (Fig. 1). To further increase the annotation functionality, all contigs were mapped to the reference canonical pathways in the Kyoto Encyclopedia of Genes and Genomes (KEGG). KEGG mapping resulted in 861 contigs that were assigned to 117 KEGG pathways, with 35.4 % of *P. ulmi* KEGG enzymes identified as transferases, 25.2 % as hydrolases, 22.4 % as oxidoreductases, 8.1 % as ligases, 6.2 % as lyases, while isomerases constituted for 2.7 % of annotated enzymes (Additional file 6). Our GO and KEGG analyses of the *P. ulmi* transcriptome are in line with previous GO analyses of *P. citri* transcriptomes where cellular process, binding and cell were the three major sub-categories [33, 34] and indicates that our approach using the Illumina HiSeq platform provided an extensive representation of the *P. ulmi* transcriptome.

### Analysis of *Panonychus* genes involved in xenobiotic resistance

In general, arthropods have developed two types of mechanisms to cope with xenobiotic compounds, both of which can contribute to the development of resistance: mechanisms that decrease exposure due to quantitative or qualitative changes in major detoxification enzymes and transporters (pharmacokinetic mechanisms) and mechanisms that decrease sensitivity due to changes in target site sensitivity caused by point mutations (pharmacodynamic mechanisms) [2, 42, 43]. The pharmacokinetic mechanism can be subdivided into three phases (I-III). In phase I detoxification enzymes such as cytochrome P450 monooxygenases (CYPs) and carboxylcholinesterases (CCEs) incorporate a nucleophilic functional group (a hydroxyl, carboxyl or amine group) into the toxic compound, resulting in a more reactive and water soluble compound. During phase II, enzymes such as UDP-glycosyltransferases (UGTs) and glutathione-S-transferases (GSTs) further increase the water solubility of the phase I metabolite by conjugation with endogenous molecules like sugars and glutathione, respectively. In Phase III, conjugates are transported out of the cell by cellular transporters, e.g., ABC transporters (ABCs).

In this study we mined the *P. ulmi* and *P. citri* transcriptomes for genes encoding known target-sites and major detoxification enzymes and transporters (CYPs, CCEs, UGTs, GSTs and ABCs) and compared them with those of *T. urticae,* for which a high-quality genomic assembly and annotation is available [21]. In addition, we also performed a phylogenetic analysis for all major detoxification gene families. It should be noted, however, that as transcriptomic data for *Panonychus* sp. is compared to genomic data for *T. urticae*, care should be taken when comparing numbers and ortho/homologues of *Panonychus* detoxification genes as recent gene duplications and genes with very low expression levels might be missed. In addition, expression of detoxification genes might also be stage-dependent. Hence, gene number differences between both *Panonychus* species should also be carefully interpreted as both *P. citri* transcriptomes were assembled using RNA-seq data from mixed stages [33, 34] while the *P. ulmi* transcriptome was based solely on RNA-seq data of adult females (see Methods).

#### Cytochrome P450 monooxygenases

**Fig. 2:**
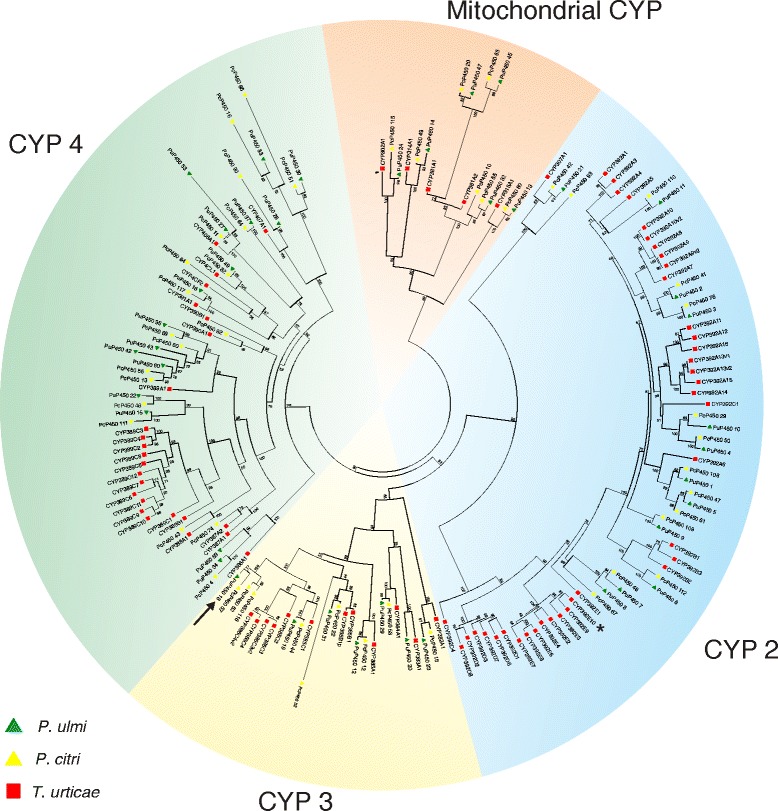
Phylogenetic analysis of *P. ulmi* putative CYPs. Maximum likelihood tree of *P. ulmi, P. citri* and *T. urticae* CYP protein sequences. The tree was constructed using MUSCLE [87] and Treefinder [89]. The tree was midpoint rooted and numbers at the branch point of each node represent the bootstrap value resulting from 1000 pseudoreplicates (LR-ELW). The scale bar represents 0.5 substitutions per site. Tetranychid CYPs clustered into the four main CYP clades: mitochondrial CYPs, CYP 2, CYP 3 and CYP 4. Colour and shape codes are as follows: *P. ulmi*, green triangle, *P. citri,* yellow triangle and *T. urticae*, red square. An arrow and an asterisk indicate CYPs associated with high levels of spirodiclofen resistance in *P. ulmi* (this study) and *T. urticae *[20], respectively. Tetranychid CYP protein sequences and accession numbers can be found in Additional file 7

Cytochrome P450 monooxygenases (P450s) are heme-containing enzymes with a diverse range of functions. Many P450s are important phase I detoxification enzymes with a crucial role in detoxification of plant secondary metabolites and in metabolizing insecticides/acaricides to less toxic compounds. Four major clans can be distinguished in the CYP gene family, namely the CYP2, CYP3, CYP4 and M (mitochondrial CYP genes) [44]. The advent of the *T. urticae* genome allowed a first insight into the CYP gene family of a phytophagous mite, revealing 81 full length CYPs. This number is similar to what is found in insects, but with an expansion of *T. urticae* specific intronless genes of the CYP2 clan [21]. A total of 63 *P. ulmi* and 118 *P. citri* CYP non-allelic ORFs could be identified (Table 1, Additional file 7). Only those CYPs that did not misalign in the final alignment and had a minimum ORF length of 450 nt were included in a phylogenetic analysis (41 *P. ulmi* and 49 *P. Citri* ORFs) (Fig. 2). Based on this analysis or based on its best BLASTx hit with a *T. urticae* CYP, all *P. ulmi and P. citri* CYPs could be assigned to one of the 4 CYP clans (Fig. 2, Table 1, Additional file 7) [21].

**Table 1:** Comparison of the CYP gene number in *T. urticae, P. ulmi* and *P. citri*

Clan	*T. urticae*	*P. ulmi* ^*a*^	*P. citri* ^*a*^
CYP 2	39	12 (16)	14 (31)
CYP 3	12	7 (8)	9 (26)
CYP 4	25	16 (30)	19 (50)
mitochondrial CYP	5	6 (9)	7 (11)
Total	81	41 (63)	49 (118)

^a^numbers without brackets represent the number of *Panonychus* CYPs that were included in phylogenetic analysis (Fig. 2) while number within brackets represent the total number of non-allelic CYP ORFs found in *Panonychus* species (see Methods)

In vertebrates, mitochondrial CYPs (clan M) are associated with steroid or vitamin D metabolism while in insects this clan comprises two groups: one group involved in the ecdysteroid metabolism pathway and another group that metabolize a variety of xenobiotic compounds [44]. We identified 9 *P. ulmi* and 11 *P. citri* ORFs in this clan and detected three clear cases of 1:1:1 orthology between *P. ulmi, P. citri* and *T. urticae*: *CYP314A1, CYP315A1* and *CYP302A1* (Additional file 7, Additional file 8, Table 1). Together with *T. urticae CYP307A1* of the CYP2 clan (see below), these CYPs are the only *T. urticae* CYPs that could be assigned as clear orthologs of insect and crustacean CYPs. They are known to be involved in the ecdysteroid pathway in insects and their tetranychid counterparts likely fulfill an analogous role [21]. Similar to *T. urticae*, orthologs of insect CYP2 clan members *CYP306A1* and *CYP18A1* could also not be identified for both *Panonychus* species (Additional file 7, Fig. 2). In insects, CYP306A1s are involved in the synthesis of 20 hydroxyecdysone (20E) by hydroxylating carbon 25, while *CYP18A1* is a hydroxylase/oxidase involved in ecdysteroid degradation [21]. Their absence in *Panonychus* sp. further corroborates that this gene cluster was lost as a whole and is an ancient trait, leading to the use of ponasterone A as the moulting hormone instead of 20E.

In insects, the number of CYP2 genes is relatively well conserved and several insect CYP2 genes are known to be involved in essential physiological functions. Compared to insects the number of CYP2 genes is more diverse in *T. urticae* and the cladoceran *Daphnia* sp. [21, 44] and at present the 392 family within the CYP2 clan is best characterized in *T. urticae*. A whole genome microarray gene expression analysis revealed that several members of the CYP2 clan were upregulated in acaricide resistant *T. urticae* strains or in a *T. urticae* strain adapted to a new challenging host plant [20, 36]. Furthermore, functional expression of two *T. urticae* CYP2 clan enzymes, CYP392A16 and CYP392E10, confirmed that these could metabolise the acaricides spirodiclofen and abamectin, respectively [20, 45]. We identified 16 and 31 CYP2 ORFs in *P. ulmi* and *P. citri* respectively. In our phylogenetic analysis, there are two clear cases of 1:1:1 orthology within the CYP2 clan (*T. urticae CYP307A1*, and *CYP392A5*). The phylogenetic analysis also suggests that the potential expansion/loss events of certain CYP2 subfamilies, like CYP392A, CYP392D and CYP392E, occurred after the split between the *Tetranychus* and *Panonychus* genus. Consequently, it was not surprising that we could not identify a clear *P. ulmi/P. citri* ortholog of *T. urticae CYP392E10* and *CYP392A16*, suggesting that spirodiclofen or abamectin metabolism must depend on different P450s or other factors in *Panonychus* species (see differential expression analysis).

Insect CYP3 and CYP4 clans comprise the insect specific families CYP4, CYP6, CYP9 and CYP325 that are well known for their involvement in environmental response/detoxification functions against xenobiotics and phytotoxins [44]. We identified 8 *P. ulmi* and 26 *P. citri* ORFs in the CYP3 clan, while 30 *P. ulmi and* 50 *P. citri* ORFs were found in the CYP4 clan. Within the CYP3 and CYP4 clan we found 5 (*T. urticae CYP382A1*, *CYP384A1*, *CYP385A1*, *CYP385B1*, *CYP385C1*) and 3 (*T. urticae CYP407A1*, *CYP391A1*, and *CYP4CL1*) clear cases of 1:1:1 orthology between *T. urticae, P. ulmi* and *P. citri*, respectively. Within the CYP4 clan, the CYP389A subfamily consists of a single gene in *T. urticae* while 4 paralogs are present in each, *P. ulmi* and *P. citri*. In contrast, the CYP389C subfamily appears to be larger in *T. urticae* compared to the two *Panonychus* species. Interestingly, Ran et al. [46] showed that *P. citri* paralogs of *T. urticae CYP389A1* were among the most up- and down regulated CYP genes in an amitraz resistant strain. Ding et al. [47], on the other hand, showed that a *P. citri* ortholog of *T. urticae CYP4CF2* was highly induced upon exposure to pyridaben while the expression of a *P. citri* ortholog of *T. urticae CYP4CL1* increased after induction by abamectin, azocyclotin, pyridaben, and spirodiclofen. In contrast to the CYP2 clan, none of these tetranychid CYP3 and CYP4 clan members have been functionally characterized.

#### Carboxylcholinesterases

Carboxylcholinesterases (CCEs) catalyse the hydrolysis of carboxylesters and have a whole gamut of physiological functions including degradation of neurotransmitters, metabolizing hormones and pheromones, regulation of behaviour and detoxification of xenobiotics [48, 49]. CCEs are phase I detoxification enzymes and their role in xenobiotic metabolism and the development of resistance in arthropods has been thoroughly studied (recently reviewed in [42]). A classification based on the CCE phylogeny, as proposed by [48] groups insect CCEs into 13 clades spread over 3 classes: the dietary/detoxification enzymes (clades A–C), the generally secreted enzymes (hormone/semiochemical processing class, clades D–G) and the neuro/developmental CCEs (clades I–M, mainly non-catalytic esterases) [48, 49]. While total numbers of CCEs in insects and *T. urticae* are similar, the neuro/developmental class is expanded in *T. urticae* with two new subclades J’ and J”. In addition, in contrast to insects no *T. urticae* CCEs have been identified within the currently recognized classification of insect dietary CCEs [21].

**Fig. 3:**
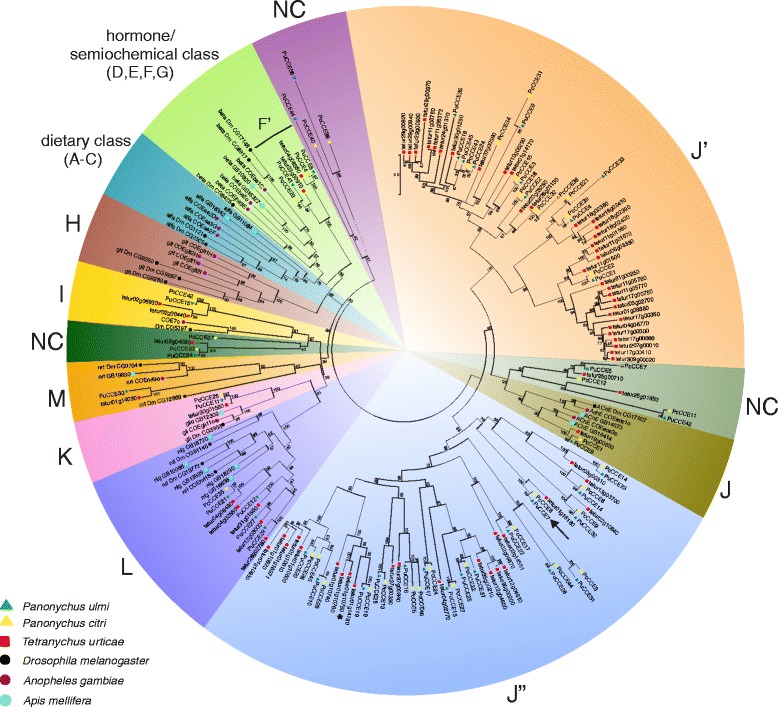
Phylogenetic analysis of *P. ulmi* putative CCEs. A set of *P. ulmi*, *P. citri*, *T. urticae, D. melanogaster, Anopheles gambiae* and *Apis mellifera* CCE protein sequences were aligned using MUSCLE, subsequently trimmed according to [49] and subjected to a maximum-likelihood analysis using Treefinder [89]. The tree was midpoint rooted and numbers at the branch point of each node represent the bootstrap value resulting from 1000 pseudoreplicates (LR-ELW). The scale bar represents 0.5 substitutions per site. Tetranychid CCEs clustered into clades [21,49]: F’ (Crustacean/Acari JhE), I, J (AChEs), J, J”, K (gliotactins), L (neuroligins), M (neurotactins) and three undetermined clades (NC). Colour and shape codes are as follows: *P. ulmi,* green triangle, *P. citri,* yellow triangle, *T. urticae,* red square, *D. melanogaster,* black dot, *Anopheles gambiae*, purple dot and *Apis mellifera*, blue dot. An arrow and an asterisk indicate CCEs associated with high levels of spirodiclofen resistance in *P. ulmi* (this study) and *T. urticae *[20], respectively. CCE protein sequences and accession numbers can be found in Additional file 9

We found a total of 61 and 91 CCE non-allelic ORFs in the *P. ulmi and P. citri* transcriptome, of which 41 and 40 ORFs of *P. ulmi* and *P. citri*, respectively, were included into a phylogenetic analysis. Based on this analysis or the best BLASTx hit with *T. urticae* CCEs, *P. ulmi and P. citri* CCEs could be assigned to one of the 13 CCE clades (Fig. 3, Table 2, Additional file 9). Similar to *T. urticae* CCEs, the majority of *Panonychus* CCEs belong to the neuro/developmental clade, with 18 *P. ulmi* and 25 *P. citri* CCEs in clade J” and 17 *P. ulmi* and 23 *P. citri* CCEs in clade J’ (Table 2, Fig. 3, Additional file 9). In sixteen cases, 1:1:1 orthologous groups could be identified between *T. urticae* and *Panonychus* species: 1 in clade J (AChE), 1 in clade K (gliotactin), 1 in clade L (neuroligins), 1 in clade J’, 2 in clade F’ (Acari/Crustacean Juvenile hormone CCEs), 3 in undetermined clades (NC) and 7 in clade J”. Remarkably, within the subclade J’, a putative expansion of CCEs in *T. urticae* (17 CCEs) was found, suggesting these *T. urticae* J’ CCEs arose after the *Tetranychus*/*Panonychus* divergence within the Tetranychidae. Finally, in clade J’ two clear *P. ulmi* orthologues of *P. citri* PcCCE16 and PcCCE39 could be identified. The latter have their best BLASTn (E-value of 0.0) hit with *P. citri* PCE1 and PCE2, respectively, which have been shown to be induced by acaricide exposure [50].

**Table 2:** Comparison of the CCE gene number in *T. urticae, P. ulmi, P. citri* and *D. melanogaster*

Class/Clade	*T. urticae*	*P. ulmi* ^a^	*P. citri* ^a^	*D. melanogaster*
Dietary class (Clade A, B, C)				13
Hormone/semiochemical class				
D				3
E				2
F				3
G				
F’	2	2	2 (4)	
Neuro/developmental class				
H				5
I	2	1	1	1
J	1	1	1	1
K	1	1	1	1
L	5	4 (13)	1 (20)	4
M	1	1	0 (4)	2
J’	34	10 (17)	10 (23)	
J”	22	16 (18)	17 (25)	
Undetermined clades (NC)	3	5 (7)	7 (12)	
Total	71	41 (61)	40 (91)	35

^a^numbers without brackets represent the number of *Panonychus* CCEs that were included in phylogenetic analysis (Fig. 3) while number within brackets represent the total number of non-allelic CCE ORFs found in *Panonychus* species (see Methods)

#### Glutathione-S-transferases

Glutathione-S-transferases (GSTs) belong to a multifunctional enzyme family, known to play an important role in phase II of xenobiotic detoxification. In addition to pesticide detoxification by conjugation, insect GSTs have also been reported to play a role in attenuation of oxidative stress caused by pesticide exposure and sporadically by metabolism of pesticides directly [51]. Insects cytosolic GSTs can be divided into seven classes [delta (δ), epsilon (ε), omega (ω), sigma (σ), theta (θ) and zeta (ζ)] with delta and epsilon GSTs well known for their role in detoxification of organophosphates and organochlorines [42, 52]. In contrast to insects, Acari also have mu-class GSTs, which were until recently thought to be verterbrate specific [21, 53].

**Fig. 4:**
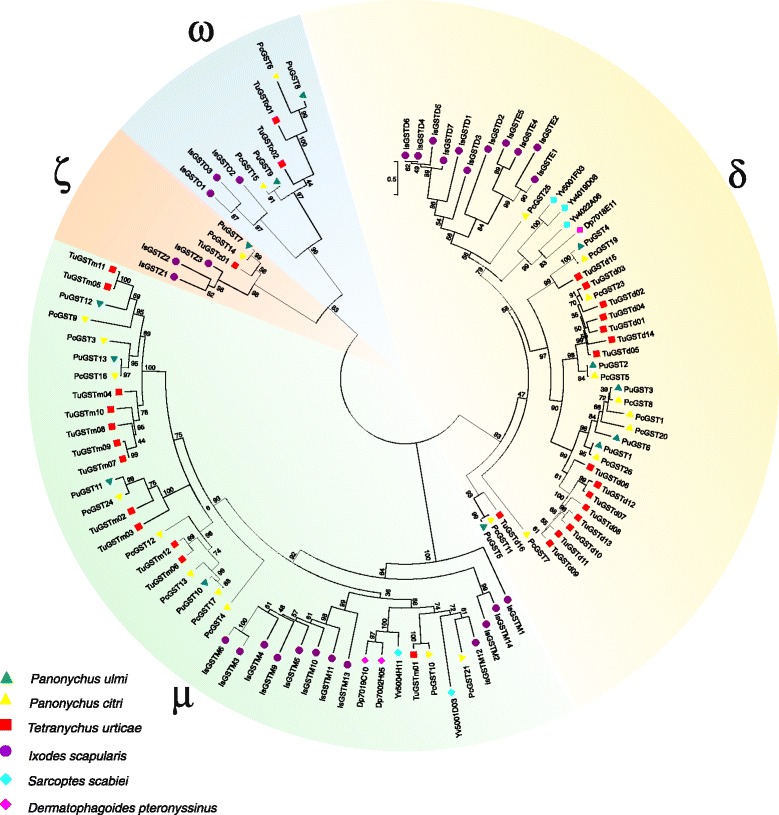
Phylogenetic analysis of *P. ulmi* putative GSTs. A set of *P. ulmi*, *P. citri*, *T. urticae, Ixodes scapularis, Sarcoptes scabiei, Dermatophagoides pteronyssinus* GST protein sequences were aligned using MUSCLE and subjected to a maximum-likelihood analysis using Treefinder. The tree was midpoint rooted and numbers at the branch point of each node represent the bootstrap value resulting from 1000 pseudoreplicates (LR-ELW). Tetranychid GSTs clustered within classes: δ, delta class, ζ, zeta class, ω, omega class and μ, mu class. Colour and shape codes are as follows: *P. ulmi,* green triangle, *P. citri,* yellow triangle, *T. urticae,* red square, purple dot, *I. scapularis*, blue rhombus, *S. scabiei* and pink rhombus, *Dermatophagoides pteronyssinus*. GST protein sequences and accession numbers can be found in Additional file 10

We found a total of 19 *P. ulmi* and 37 *P. citri* GST non-allelic ORFs of which 13 *P. ulmi* and 23 *P. citri* GSTs were included in a phylogenetic analysis (Table 3, Fig. 4 Additional file 10). Based on this analysis or based on the best BLASTp hit with *T. urticae* GSTs, *P. ulmi* and *P. citri* GSTs could be assigned to 4 GST-classes: delta, mu, omega and zeta. The number of *P. ulmi* GSTs is lower than those observed in *P. citri, T. urticae* and *Ixodes scapularis* (Table 3). Although BLASTp searches against the non-redundant database performed by [34] identified representatives of sigma and theta GST we were unable to find neither of those two classes in the transcriptome of *P. ulmi*. The lack of theta and sigma GSTs seems to be a common feature of Acari species analysed to date [53, 54]. Similar to the phylogenetic analysis of tetranychid CYP genes (see above) only limited orthologous relationships between tetranychid GSTs could be established, as illustrated by the presence of *T. urticae* specific clusters of delta and mu GSTs. In both the omega and zeta class we identified a 1:1:1 orthology between *Panonychus* contigs and *T. urticae* GSTs (*TuGSTo01*: PcGST6: PuGST8 and *TuGSTz01*: PcGST14: PuGST7). Within the mu GSTs we found two 1:1:1 orthologs (*TuGSTm11/TuGSTm05*: PuGST12: PcGST9 and *TuGSTm02*: PcGST24: PuGST11) while no 1:1:1 orthologs could be identified in the delta GSTs (Fig. 4).

**Table 3:** Comparison of the cytosolic GST gene number in *T. urticae, P. ulmi, P. citri* and *I. scapularis*

Class	*T. urticae* ^*a*^	*P. ulmi* ^*b*^	*P. citri* ^*b*^	*I. scapularis* ^*c*^
delta	16	6 (11)	10 (16)	12^*d*^
mu	12	4 (5)	10 (18)	14
omega	2	2	2	3
zeta	1	1	1	3
Total	31	13 (19)	23 (37)	32

^*a*^in [21], the number of theta and zeta GSTs was switched: it should be zero theta *T. urticae* GSTs and one zeta *T. urticae* GST

^*b*^numbers without brackets represent the number of *Panonychus* GSTs that were included in phylogenetic analysis (Fig. 4) while number within brackets represent the total number of non-allelic GST ORFs found in *Panonychus* species (see Methods)

^*c*^numbers derived from Reddy et al. [53]

^*d*^the number of *I. scapularis* epsilon (5) and delta GSTs (7) was merged as it was not clearly shown in the *I. scapularis* GST gene family study by Reddy et al. [53] that epsilon GSTs actually do cluster with epsilon GSTs of insects

Using a whole genome gene-expression microarray, members of the same GST classes were also shown to be highly upregulated in multi-resistant *T. urticae* strains [36]. Three GST genes (*TuGSTd10*, *TuGSTd14*, *TuGSTm09*) that were upregulated in a strain MAR-AB highly resistant against abamectin and other important acaricides were also functionally expressed. Out of these, TuGSTd14 showed the highest affinity toward abamectin and had a competitive type of inhibition, suggesting the acaricide may bind to the H-site of the enzyme [55]. However, as mentioned earlier no clear *Panonychus* orthologs of *TuGSTd14* could be identified in our study.

#### ABC transporters

The ATP Binding Cassettte (ABC) protein family is a large and ubiquitous family of proteins. Most members of this family use ATP to transport substrates across lipid membranes, and hence are referred to as ABC transporters. Based on the sequence similarity of their nucleotide binding domain (NBD, the domain that binds ATP) the ABC protein family can be divided into eight subfamilies (A to H). Although extensively characterized for their involvement in drug resistance in vertebrates, the role of ABC transporters in arthropod xenobiotic resistance is less well known. Nevertheless, ABC transporters have been associated with resistance to as many as 27 insecticides/acaricides from nine different chemical classes [56]. However, the major source of evidence linking these arthropod ABC transporters to resistance has been ABC transporter expression quantification or synergism studies [56]. A total of 103 ABC genes were identified in the *T. urticae* genome, the highest number discovered in a metazoan species to date. This high number of ABC genes in *T. urticae* is due predominantly to lineage-specific expansions of the ABCC, ABCG and ABCH subfamilies [57].

**Fig. 5:**
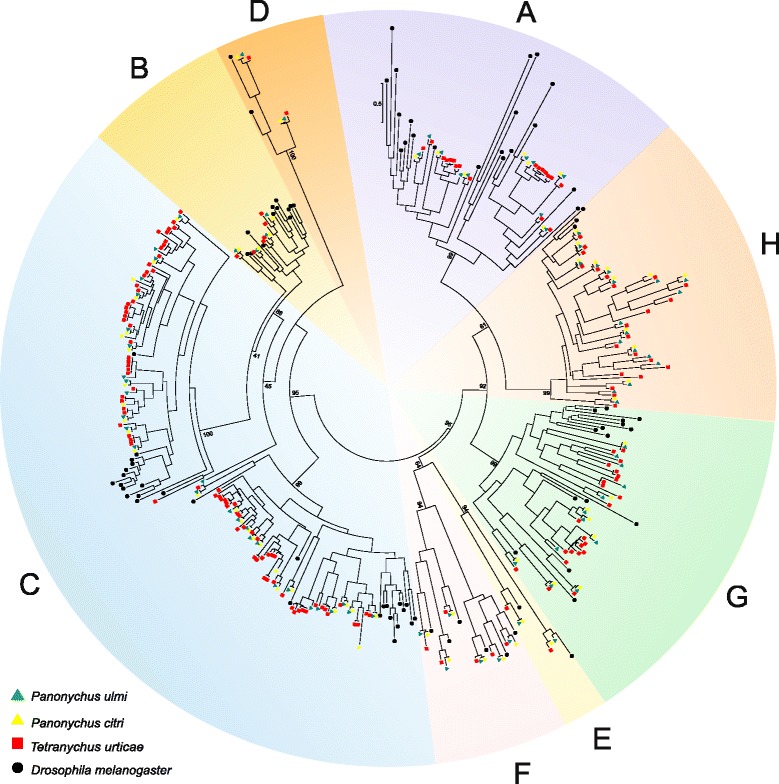
Phylogenetic analysis of *P. ulmi* ABC protein NBDs. Maximum likelihood midpoint rooted tree of N- and/or C-terminal NBDs extracted from *P. ulmi* and *P. citri* ABC protein coding ORFs and *T. urticae* and *D. melanogaster* ABC protein sequences. The scale bar represents 0.5 amino-acid substitutions per site. *P. ulmi* and *P. citri* NBDs clustered within the eight currently described ABC subfamilies (**a** to **h**). The tree was midpoint rooted and numbers at the branch point of each node represent the bootstrap value resulting from 1000 pseudoreplicates (LR-ELW). C-terminal NBDs of ABCC transporters clustered together with NBDs of ABCB transporters [57]. Colour and shape codes are as follows: *P. ulmi,* green triangle, *P. citri,* yellow triangle, *T. urticae,* red square and *D. melanogaster,* black dot. ABC protein sequences and accession numbers can be found in Additional file 11

We found 145 *P. ulmi* and 230 *P. citri* ORFs with a tBLASTn hit with *T. urticae* ABC protein sequences (Additional file 11). NBDs from the ORFs of these contigs, when present, were extracted and used in a maximum likelihood phylogenetic analysis. Similar to previous studies [57] C-terminal NBDs of ABCC transporters clustered together with NBDs of ABCB transporters (Fig. 5, Additional file 11). Based on this analysis or, in case the ORF did not encode an NBD, based on its best BLASTx hit with *T. urticae* ABC proteins, we assigned *P. ulmi* and *P. citri* ABC ORFs to one of the 8 ABC gene subfamilies (Table 4, Fig. 5, Additional file 11). As ABC genes are large genes (around 2 to 5 kb in size), they can be easily fragmented into multiple contigs assembled from RNA-seq reads. This could result in an overestimation of the number of putative ABC genes. Therefore, we used the number of NBDs to estimate quantitative differences among the ABC gene families in the Tetranychidae (Table 4). The number of *T. urticae* NBDs is much higher than the number of *P. citri* and *P. ulmi* NBDs, and is mainly due to a higher number of *T. urticae* NBDs in the ABCA, ABCC, ABCG and ABCH gene subfamily. In several cases *T. urticae* ABCH NBDs do not have a counterpart in *Panonychus* species while in both the ABCA, ABCC as ABCG gene family a putative expansion of *T. urticae* NBDs compared to *Panonychus* NBDs can be suspected. Interestingly, for exactly two of these *T. urticae* ABC gene subfamilies it was shown that several genes were differentially expressed in multi-pesticide resistant strains and/or in mites transferred to challenging host plants [36]. Finally, NBDs of ABC transporters that are considered as conserved in arthropods (ABCB half transporters (*D. melanogaster CCG7955*, *CG1824*), ABCC (*D. melanogaster CG7806*, *sur*), ABCD, ABCE, ABCF, ABCG (*D. melanogaster CG3327*, *CG11069*, *CG31121*)) were also found in both *Panonychus* species (Table 4, Fig. 5) [56].

**Table 4:** Comparison of the number of *P. ulmi*, *P. citri* and *T. urticae* ABC protein NBDs

Class	*T. urticae*	*P. ulmi*	*P. citri*
A	18	10	7
B	6	4	4
C	78	28	26
D	2	2	2
E	2	2	2
F	6	6	6
G	23	12	8
H	22	18	12
Total	157	82	67

### Horizontally transferred genes

Horizontal gene transfer (HGT) refers to the asexual transfer of genetic information between non-related species. In bacteria and fungi HGT is a strong evolutionary force contributing to adaptation and colonization of challenging environments. Due to the unique characteristics of animal biology, the prevalence and impact of HGT on animal evolution was considered to be minor. However, recent studies have uncovered a vast array of horizontally transferred genes (HTGs) in the *T. urticae* genome with plausible roles in xenobiotics detoxification or other responses to the environment. These include 80 UDP-glycosyltransferases [39], 16 intradiol ring-cleavage dioxygenases [36], 3 carotenoid desaturases, 2 carotenoid cyclase/synthases [21, 35], 2 levanases [21], a cyanate lyase [37], β-cyanoalanine synthase [38] and a methionine synthase [21]. Orthologs of these *T. urticae* HTGs were identified in both *Panonychus* transcriptomes (Table 5 and 6, Additional file 12). The presence of these genes in the *Panonychus* genus strongly suggests that these horizontal gene transfers occurred before the split of the *Tetranychus* and *Panonychus* genus. As the majority of the microbial xenologues of these unique spider mite genes code for enzymes able to detoxify and break down plant secondary metabolites, it has been argued that HGT facilitated spider mite adaptations to a phytophagous lifestyle [38]. In the sections below we will discuss some of these tetranychid HGT families more into detail.

**Table 5:** Comparison of the UGT gene number in *T. urticae, P. ulmi, P. citri* and *I. scapularis*

Family	*T. urticae*	*P. ulmi* ^*a*^	*P. citri* ^*a*^
201	36	9 (10)	12 (23)
202	17	3 (5)	6 (8)
203	11	4 (5)	5 (9)
204	8	3	3
205	6	4 (7)	4 (7)
206	1	1 (2)	1
207	1	1	1
Total	80	25 (33)	32 (52)

^*a*^numbers without brackets represent the number of *Panonychus* UGTs that were included in phylogenetic analysis (Fig. 6) while number within brackets represent the total number of non-allelic UGT ORFs found in *Panonychus* species (see Methods)

**Table 6:** Putative horizontally transferred genes identified in the *T. urticae* genome and *Panonychus* spp. transcriptomes

	Carotenoid desaturase^1^	Carotenoid cyclase/synthase	Cyanate lyase	Cysteine synthase A^2^	IDRCD^3^	Levanase^4^	Methionine synthase^5^
*T. urticae*	3	2	1	1	17	2	1
*P. ulmi* ^*a*^	2 (3)	2	1	1 (2)	8 (12)	2	1
*P. citri* ^*a*^	3 (6)	2 (4)	1 (2)	1 (2)	16 (23)	2 (5)	1 (5)

^*a*^numbers within brackets show the total number of ORFs for each family of HTGs (excluding UGTs, see Table 5). Numbers without brackets depict HTG ORFs coding for proteins with an amino acid (AA) sequence length ≥ than 150 AA (carotenoid desaturases, levanases and methionine synthase), ≥ 200 AA (carotenoid synthases) and ≥ 100 AA (cyanate lyase and IDRCDs)

#### UDP-glycosyltransferases

UDP-glycosyltransferases (UGTs) are common in the majority of living organisms including viruses, bacteria, plants and animals. They catalyze the conjugation of small lipophilic molecules with uridine diphosphate (UDP) sugars, increasing their water solubility. As such, UGTs play an important role in the synthesis, storage and transport of secondary metabolites. In vertebrates, UGTs are also well studied because of their role in phase II drug metabolism [39, 58]. Although a role for UGTs in arthropod xenobiotic resistance was suggested more than twenty years ago [59], only recently has functional evidence for their role in xenobiotic resistance been presented [60–63]. It has been suggested that the UGT gene family might have been lost early in the Chelicerata lineage and subsequently re-gained in the tetranychid mites by means of the horizontal gene transfer from bacteria. This discovery provides important clues to UGTs functions in relation to detoxification and therefore host adaptation in the phytophagous mites.

**Fig. 6:**
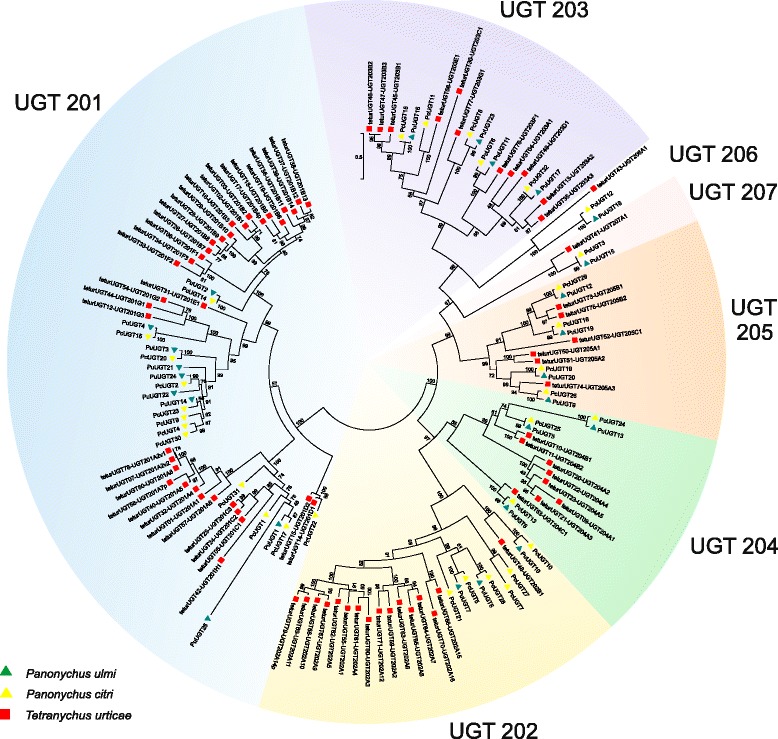
Phylogenetic analysis of *P. ulmi* putative UGTs. Maximum likelihood midpoint rooted tree of *P. ulmi*, *P. citri* and *T. urticae* UGT protein sequences. The scale bar represents 0.5 amino-acid substitutions per site. Numbers at the branch point of each node represent the bootstrap value resulting from 1000 pseudoreplicates (LR-ELW). Color and shape codes are as follows: *P. ulmi,* green triangle, *P. citri,* yellow triangle and *T. urticae,* red square. UGT protein sequences can be found in Additional file 12

We identified a total of 33 and 52 *P. ulmi* and *P. citri* UGT non-allelic ORFs, respectively. A subset of 24 *P. ulmi* and 32 *P. citri* ORFs were included in a phylogenetic analysis (Fig. 6). Based on this analysis or based on the *T. urticae* best BLASTx hit, *P. ulmi* and *P. citri* UGTs could be assigned to one of the UGT subfamilies (Fig. 6, Table 5, Additional file 12). Within Tetranychidae the UGT201 subfamily is the largest UGT subfamily with 10, 23 and 36 UGT ORFs/genes in *P. ulmi*, *P. citri* and *T. urticae*, respectively (Table 5, Additional file 12). The UGT201A, UGT201B, UGT202A and UGT204A subfamily are clearly more numerous in *T. urticae* compared to both *Panonychus* species while the opposite could be observed for the UGT201G subfamily, suggesting the UGTs in these subfamilies probably arose in *T. urticae* or were lost in *P. ulmi* after diversification within the Tetranychidae. Finally, within different UGT subfamilies clear 1:1:1 orthologous relationships could be observed between *Panonychus* UGTs and *T. urticae UGT202B1*, *UGT203G1*, *UGT203F1*, *UGT204C1*, *UGT205A3*, *UGT206A1* and *UGT207A1* (Fig. 6).

#### Intradiol ring-cleavage dioxygenases (IDRCDs)

**Fig. 7:**
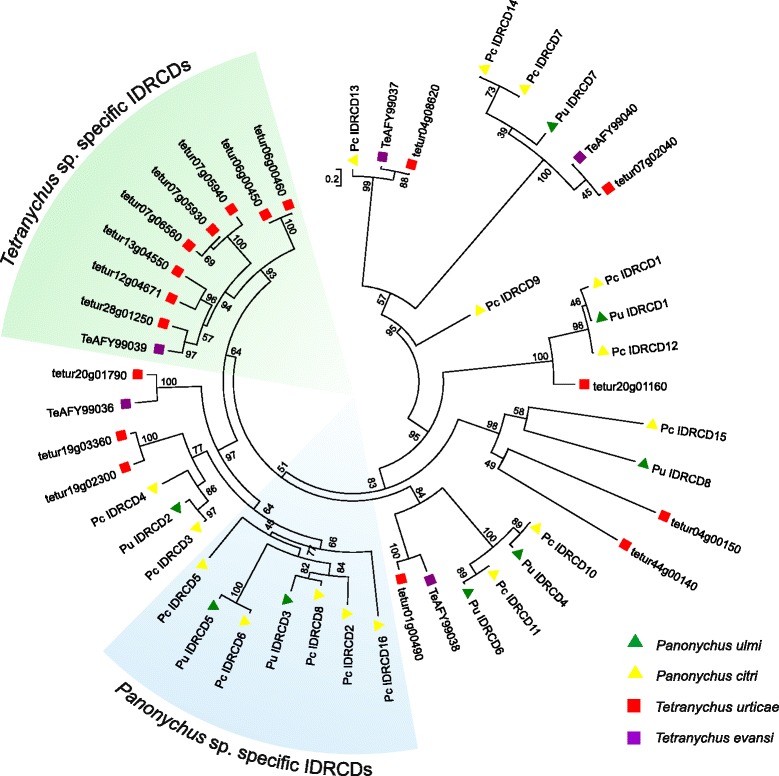
Phylogenetic analysis of *P. ulmi* putative IDRCDs. Maximum likelihood midpoint rooted tree of *P. ulmi*, *P. citri*, *T. urticae* and *T. evansi* IDRCD protein sequences. The scale bar represents 0.2 amino-acid substitutions per site. Numbers at the branch point of each node represent the bootstrap value resulting from 1000 pseudoreplicates (LR-ELW). *Panonychus* sp. and *Tetranychus* sp. specific clades are shaded in blue and green, respectively. IDRCD protein sequences and accession numbers can be found in Additional file 12

Among Metazoa, tetranychid mites are the only species known to harbor genes encoding IDRCDs. In bacteria and fungi these enzymes catalyze the oxygenolytic fission of catecholic substances, allowing them to degrade aromatic rings, an essential step in the carbon cycle [21, 36]. However, their role in tetranychid mites has not yet been characterized. About half of the number of genes in this family were differentially expressed in mites upon host plant change and in multi-resistant *T. urticae* strains, and their expression patterns were highly correlated, suggestive of a role for this gene family in xenobiotic resistance [36]. Twelve *P. ulmi* and 23 *P. citri* contigs showed a tBLASTn hit with *T. urticae* IDRCD proteins. A phylogenetic analysis revealed several orthologous relationships between tetranychid IDRCDs (*tetur01g00490*, *tetur04g00150*, *tetur04g08620*, *tetur07g02040*, *tetur10g00490*, *tetur19g02300*, *tetur20g01160*), suggesting these IDRCD genes arose before the split between the *Tetranychus* and *Panonychus* genus (Fig. 7). Furthermore, an expansion of IDRCDs can be observed in both *T. urticae* (17 IDRCD genes) as in *P. ulmi and P. citri* (12 and 23 genes respectively). Remarkably, exactly those IDRCDs that are absent in both *Panonychus* species are highly upregulated in mites upon host plant change and in multi-resistant *T. urticae* strains [36].

#### Other horizontal gene transfers

Besides UGTs and IDRCDs we also identified *Panonychus* orthologs of *T. urticae* carotenoid desaturases, carotenoid cyclases, levanases, cyanate lyase, β-cyanoalanine synthase and methionine synthase (Table 6, Additional file 12). Wybouw et al. [38] showed that a *T. urticae* strain overexpresses β-cyanoalanine synthase when adapted to cyanogenic plants. Functional expression also revealed that this enzyme is able to detoxify cyanide which is the main defensive phytochemical of these plants. In contrast to *T. urticae, Panonychus* sp. almost exclusively feed on Rosaceae [64], a cyanogenic plant family producing cyanide in various tissues as plant defense [65]. As such, β-cyanoalanine synthase might have been crucial in conferring resistance in *Panonychus* sp. to this continuous exposure to dietary cyanide.

### Target-sites of insecticides/acaricides

Since the PSR-TK strain originates from the field and has evolved resistance to at least hexythiazox, clofentezine and spirodiclofen [17], the *P. ulmi* transcriptome was mined for contigs encoding known target sites of acaricides, in order to search for SNPs associated with target-site resistance. We successfully annotated acetylcholinesterase (AChE), voltage gated sodium channel (VGSC), GABA- and glutamate-gated chloride channels (Rdl and GluCl), chitin synthase 1 (CHS1), acetyl-CoA carboxylase (ACCase) and cytochrome B (cytB) [6, 23, 24, 42, 66–72] (see Table 7 for an overview and the Insecticide Resistance Action Committee (IRAC) mode of action classes that target these proteins [41]). To obtain *P. ulmi* target-site sequences as full-length as possible, we also mined an alternative *P. ulmi* assembly, which was constructed using the Velvet/Oases package (Additional file 13), and combined contigs from the latter assembly with the one described in this study (CLC Genomics Workbench).

**Table 7:** Known target sites of acaricides, their *T. urticae* gene ID and presence in *P. ulmi* and the IRAC acaricide classes that target these proteins

Target site	*T. urticae* ^*a*^		*P. ulmi* ^*b*^	IRAC group	Reference
	gene ID	length (nt)	length (nt)		
VGSC	*tetur34g00970*	6600	6696	3 (Sodium channel modulators)	[66, 69]
AChE	*tetur19g00850*	2064	2193	1 (AChE inh.)	[70, 72]
ACCase	*tetur21g02170*	6957	6594	23 (Accase inh.)	[6]
cytB	*FJ196444*	1063	1041	20 (Mt Complex III inh.)	[67, 68]
CHS1	*tetur03g08510*	4608	4170	10 (Mite growth inh.)	[22, 24]
GluCl1	*tetur02g04080*	1338	1335	6 (Cl channel activators)	[23, 71]
GluCl2	*tetur08g04990*	1368	987		
GluCl3	*tetur10g03090*	1629	1623		
GluCl4	*tetur22g02450*	1325	1344		
GluCl5	*tetur36g00090*	1392	1317		
GluCl6	*tetur41g00120*	1392	-		
Rdl1	*tetur12g03620*	2346	2049		
Rdl2	*tetur36g00580*	1572	1608		
Rdl3	*tetur36g00590*	2187	2019		

^*a*^ *T. urticae* gene IDs can be accessed at the ORCAE database (http://bioinformatics.psb.ugent.be/orcae/overview/Tetur) while FJ96444 can be accessed at the NCBI database

^*b*^ *P. ulmi* target site sequences can be found in Additional file 14

Compared to insects *T. urticae* has a higher number of Rdl and GluCl genes, with the majority of insects having only one Rdl and GluCl gene while *T. urticae* has 3 and 6, respectively [23]. Similar to *T. urticae* we identified 5 *P. ulmi* GluCl and 3 Rdl genes (Additional file 14), suggesting that the Rdl and GluCl gene family diversified before the radiation of the Tetranychidae. Next, we compared all known target-site sequences of the spirodiclofen susceptible (HS) and resistant (PSR-TK) *P. ulmi* strains to identify non-synonymous single nucleotide polymorphisms (SNPs) that are unique for the resistant strain. For AChE, we identified the F331W substitution (*Torpedo californica* numbering) that was fixed in the PSR-TK strain but segregating in the HS strain. In the past, it has been shown that a substitution at this position causes resistance to organophosphate (OP) and carbamate (CB) insecticides/acaricides [42] and the presence of this substitution in both *P. ulmi* strains emphasizes the scope of selection exerted by OPs and CBs during the second half of the 20th century.

Comparing ACCase sequences of both strains revealed several non-synonymous fixed SNPs in the PSR-TK strain that were not present in the susceptible HS-strain. At present, only one ACCase substitution has been associated with resistance against cyclic keto-enols: an E645K substitution in the whitefly *Trialeurodes vaporariorum* [73]. However, the residue is not conserved in mites and at the corresponding position in the *P. ulmi* ACCase protein, the sequence is identical in both strains (a glutamine; at position 566, *T. urticae* numbering *tetur21g02170*). Recently, it has been shown that the activated enol derivative of the insecticide spirotetramate interacts with the carboxyltransferase domain of ACCase [6], suggesting this region as a prime site to expect resistance mutations. Within this domain region, we also found several residues that were different between the ACCase protein sequence of the PSR-TK and the HS-strain (Additional file 14, Additional file 15). However, it is unclear whether these substitutions play a role in resistance. Similarly to ACCase, other target site sequences (VGSC, cytB, GluCl, Rdl and CHS1) contained non-synonymous SNPs in the PSR-TK strain that were not present in the HS strain, but none were located at positions previously reported to be involved in acaricide resistance [42].

### Differential expression analysis between an acaricide resistant (PSR-TK) and susceptible strain (HS) of *P. ulmi* using RNAseq data

**Fig. 8:**
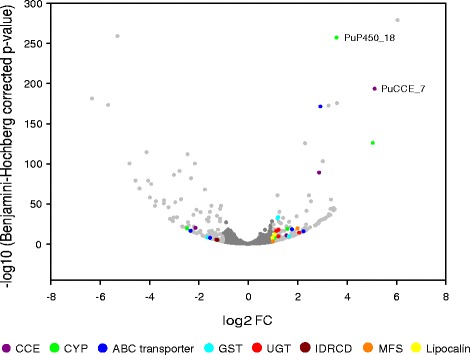
Volcano plot of differentially expressed contigs in the acaricide resistant PSR-TK strain relative to the susceptible HS strain. The negative log_10_ of Benjamini-Hochberg (BH) adjusted *p*-values was plotted against the log_2_FC in gene expression. *P. ulmi* contigs up- and downregulated, by twofold or more (123 and 122 genes, respectively) are depicted as light grey circular dots. Circular dots of various colours depict contigs with a best BLASTx hit with *T. urticae* proteins putatively involved in xenobiotic detoxification ([36], see also Additional file 17). Contigs encoding detoxification genes putatively involved in spirodiclofen resistance are indicated in the plot by ID PuP450_18 (contig_01016, log_2_(FC) = 5.1, BH adjusted p-value = 1,1E^−193^) and ID PuCCE_7 (contig_00577, log_2_(FC) = 5.1, BH adjusted p-value =1,4E^−257^)

**Fig. 9:**
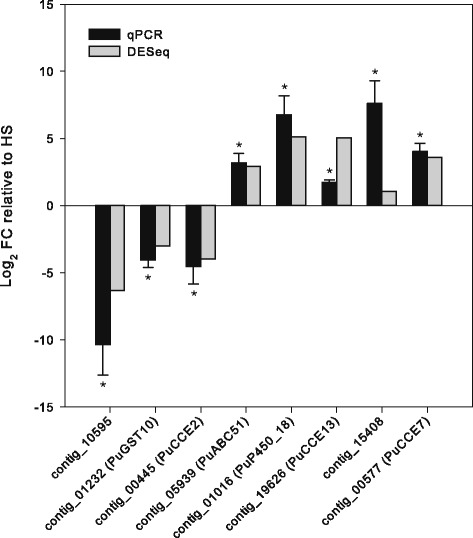
Validation of differentially expressed *P. ulmi* contigs by qPCR. Five upregulated and three downregulated contigs determined by differential gene expression analysis of RNA-seq data (see Fig. 8, Additional file 17) were selected for qPCR analysis. The data from qPCR were presented as mean of three replicates. Error bars represent the standard error of the calculated mean. Asterisks indicate significantly different expression values compared to the reference condition (HS)

RNA was extracted from 200 1–3 day old adult female *P. ulmi* mites from the PSR-TK and HS strains with four-fold biological replication (see Methods). Using the replicated RNAseq data and the DESeq2 software, we performed a differential expression analysis between the acaricide resistant (PSR-TK) and a susceptible (HS) strain of *P. ulmi*. Only those contigs that (1) were not considered as contamination by the NCBI contamination screen (2) have strand-specific reads and (3) have a BLASTx hit were included in our differential expression analysis (see Methods for more details). For all *P. ulmi* contigs the number of mapped reads per contig can be found in Additional file 16. For further analyses described below, we excluded *P. ulmi* contigs without a BLASTx hit as a biological role of the proteins coded by these contigs cannot be assigned. We observed that 123 of 8722 *P. ulmi* contigs were significantly upregulated (Benjamini-Hochberg adjusted *p*-value ≤ 0.05 and │FC│ > 2) in the PSR-TK strain compared to the HS strain, while 122 were significantly downregulated (Fig. 8, Tables 8 and 9, Additional file 17). For a subset of contigs (genes), the differential expression analysis based on RNAseq was validated by qPCR on cDNAs of the same populations (Fig. 9).

**Table 8:** Top 20 of upregulated contigs in the acaricide resistant *P. ulmi* PSR-TK strain relative to the susceptible HS strain. See Additional file 17 for a full list of differentially expressed contigs

Contig ID^a^	log2FC	Blast2GO description	*T. urticae* BLASTx hit (E-value)^b^	*T. urticae* gene name
**Contig_00184**	6,06	RNA polymerase	-	-
*Contig_01016*	5,13	cytochrome family	*tetur26g01470* (0)	cytochrome P450 monooxygenase (*CYP385C1*)
Contig_19626	5,06	acetylcholinesterase	*tetur35g00210* (3,00E^−171^)	Carboxylcholinesterases (*TuCCE66*)
Contig_08154	3,61	farnesoic acid o-methyl transferase-like protein	*tetur30g00770* (3,00E^−64^)	farnesoic acid O-methyltransferase-like
*Contig_00577*	3,59	acetylcholinesterase	*tetur01g16180* (0)	Carboxylcholinesterases (*TuCCE13*)
**Contig_14289**	3,52	-	*tetur30g01870* (7,00E^−17^)	hypothetical protein
Contig_09906	3,51	collagen alpha-4 chain	*tetur38g00240* (1,00E^−23^)	collagen alpha-2
Contig_21107	3,47	acetyl- partial	*tetur21g02170* (2E^−22^)	ACCase
Contig_01487	3,51	cathepsin partial	*tetur25g00650* (2,00E^−95^)	cathepsin L
Contig_14168	3,47	adenylyl cyclase associated protein	*tetur14g00690* (2E^−30^)	adenylate cyclase-associated CAP
Contig_00198	3,36	polyprotein	-	-
Contig_08809	3,34	RNA polymerase 1	*tetur02g08750* (3,00E^−09^)	RNA-directed RNA polymerase 1
**Contig_04691**	3,28	polyprotein	-	-
Contig_06838	3,27	glucosylceramidase isoform 2	*tetur02g12930* (0)	glycoside hydrolase
Contig_26919	3,27	intermediate in toll signal transduction	*tetur03g05570* (2,00E^−27^)	evolutionarily conserved signaling intermediate in Toll pathway
Contig_26878	3,17	chimeric r1 r2 retrotransposon	*tetur04g02090* (2,00E^−26^)	similar to gag-like protein
Contig_12550	3,10	collagen alpha-1	*tetur01g04240* (3,00E^−18^)	collagen alpha-1
Contig_12959	3,07	n/a	*tetur03g05210* (2,00E^−17^)	U6 snRNA phosphodiesterase isoform X1 putative
Contig_16990	3,03	leucine-rich repeat	*tetur14g01680* (0)	similar to leucine-rich transmembrane protein
Contig_25316	3,00	RNA polymerase 2-like	*tetur02g08750* (2,00E^−15^)	RNA-directed RNA polymerase 1

^a^contigs indicated in bold have their best BLASTx hit with biRNA viruses while contigs indicated in italic contain a gene of which the upregulation is the result of spirodiclofen selection

^b^E-value threshold was set at 1E^−5^

**Table 9:** Top 20 of downpregulated contigs in the acaricide resistant *P. ulmi* PSR-TK strain relative to the susceptible HS strain. See Additional file 17 for a full list of differentially expressed contigs

Contig ID^a^	log2FC	Blast2GO description	*T. urticae* BLASTx-hit (E-value)^b^	*T. urticae* gene name
Contig_10595	−6,31	nucleolin 2-like	*tetur04g03300* (9,00E^−17^)	4Fe-4S ferredoxin, iron-sulpur binding domain
Contig_03877	−6,05	krab-a domain-containing protein	*tetur03g07110* (7,00E^−05^)	zinc finger protein
Contig_04800	−5,66	chemosensory gustatory receptor family	*tetur07g05030* (5,00E^−25^)	gustatory receptor family
Contig_06558	−5,29	DNA polymerase	*tetur21g01960* (6,00E^−09^)	pacifastin
Contig_02213	−4,80	histone-lysine n-methyltransferase setmar-like	*tetur04g09537* (8,00E^−06^)	transposable element Tc3-related transposase
**Contig_04690**	−4,55	polyprotein	-	-
Contig_09905	−4,39	collagen alpha-4 chain	*tetur38g00240* (9,00E^−24^)	collagen alpha-2
Contig_03088	−4,10	farnesoic acid o methyltransferase-like protein	*tetur30g00780* (1,00E^−14^)	farnesoic acid O-methyltransferase-like protein
Contig_02422	−4,04	reverse transcriptase maturase	*tetur21g01960* (3,00E^−11^)	proteinase inhibitor I19, pacifastin
*Contig_00445*	−3,99	esterase type b	t*etur20g03250* (0)	Carboxylcholinesterases (*TuCCE50*)
Contig_02182	−3,97	retrovirus-related pol polyprotein from transposon partial	*tetur09g03700* (1,00E^−18^)	hypothetical cell surface protein
Contig_08551	−3,92	adenylyl cyclase-associated protein	*tetur14g00690* (1,00E^−29^)	adenylate cyclase-associated CAP
Contig_08025	−3,76	Acetyl - partial	*tetur21g02170* (1,00E^−29^)	ACCase
Contig_10729	−3,73	hypothetical protein TcasGA2_TC016169	*tetur04g02090* (3,00E^−17^)	similar to gag-like protein
Contig_09937	−3,41	expressed sequence ai451617	*tetur03g07110* (3,00E^−12^)	zinc finger protein
Contig_04137	−3,40	alpha-galactosidase a	*tetur02g04310* (1,00E^−42^)	glycoside hydrolase, catalytic core
Contig_08881	−3,21	reverse transcriptase	*tetur63g00020* (6,00E^−18^)	hypothetical protein
**Contig_00197**	−3,16	structural polyprotein	-	-
Contig_11839	−3,07	paired box and transposase domain	*te*tur04g09537 (2,00E^−13^)	transposable element Tc3-related transposase
Contig_00506	−3,04	retrovirus-related pol	*te*tur40g00380 (4,00E^−14^)	hypothetical protein

^a^contigs indicated in bold have their best BLASTx hit with *D. melanogaster* biRNA viruses while the contig indicated in italic contains a CCE gene of which the downregulation is the result of spirodiclofen selection

^b^E-value threshold was set at 1E^−5^

Several up- and downregulated contigs showed high homology with sequences of arthropod bisegmented double stranded RNA viruses (biRNA viruses) (Tables 8 and 9, Additional file 17). There are several reports documenting viruses infecting *Panonychus* species [74–77]. As such, differential expression of biRNA viruses between the two resistant strains might reflect the different virus composition and infection status*.*

Among the differentially expressed contigs, 29 coded for major detoxification enzyme families or potential new players in detoxification (see above and [36]). Among the upregulated contigs, we identified 4 CCEs, 3 CYPs, 3 GSTs, 3 ABCs, 4 UGTs, 2 lipocalins and 2 Major Facilitator Superfamily genes while 1 CYP, 2 CCEs, 1 GST, 2 ABCs and 2 IDRCDs were downregulated (Fig. 8, Additional file 17). Such a broad response in detoxifying enzyme families is typically also encountered in the spider mite *T. urticae*, where numerous gene-expression studies of susceptible and resistant strains have shown an overlap in the gene-families involved [20, 36, 78], but also specificity in term of the members of these families.

**Fig. 10:**
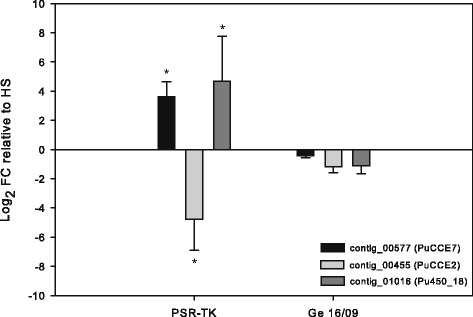
Effect of spirodiclofen selection on expression of genes putatively involved in spirodiclofen resistance. qPCR quantification of expression levels of contig_01016 (PuP450_18), contig_00577 (PuCCE_7) and contig_00445(PuCCE_2) in *P. ulmi* strains HS (spirodiclofen susceptible strain), PSR-TK (spirodiclofen resistance ratio > 7000 relative to HS) and Ge 16/09, the parental strain of PSR-TK (spirodiclofen resistance ratio of 59 relative to HS) [17]. The data from qPCR were presented as mean of three replicates. Error bars represent the standard error of the calculated mean. Asterisks indicate significant different expression values compared to the reference condition (HS)

Two differentially expressed contigs also coded for the same small (~ 200 bp) fragment of the gene coding for ACCase, the target-site of cyclic keto-enols [6] (see above), but with a six nucleotide difference between the two contig sequences. One of these contigs was highly upregulated (contig_21107) in the PSR-TK strain according to our analysis while the other was highly downregulated (contig_08025). A close inspection of mapped reads revealed that sequences polymorphism could explain this result (see Additional file 16). Therefore, we mapped all reads from both *P. ulmi* strains against the manually assembled *P. ulmi* ACCase gene (see above) and found no significant difference in expression (data not shown). Two contigs encoding CCEs (contig_19626/PuCCE13 and contig_00577/PuCCE7) and a CYP (contig_01016/PuCYP_18) were among the most highly upregulated contigs while another CCE contig (contig_00445/PuCCE2) was one of the most downregulated (Fig. 9, Tables 8 and 9, Additional file 17). RT-qPCR data confirmed the upregulation of contig_00577/PuCCE7 and contig_01016/PuCYP18 and the downregulation of contig_00445/PuCCE2, while upregulation of contig_19626/PuCCE13 was only partially confirmed (Fig. 9). qPCR expression analysis also confirmed that the up- and downregulation of contig_00577/PuCCE7 and contig_00445/PuCCE2 in PSR-TK might be the result of spirodiclofen selection, with both contigs being slightly downregulated in Ge 16/09 (parental strain of PSR-TK) and highly up- and downregulated in PSR-TK relative to HS, respectively (Fig. 10). In addition, the same pattern was found for a CYP (contig_01016/PuP450_18) which is highly upregulated in PSR-TK-while and slightly downregulated in GE 16/09 compared to the HS strain (Fig. 10).

Previously, it was shown that the P450 inhibitor PBO and the esterase inhibitor DEF could enhance the toxicity of spirodiclofen in a highly spirodiclofen resistant *T. urticae* strain [18] and in a spirodiclofen resistant *P. citri* strain [19]. Similarly, Kramer and Nauen [17] showed that PBO enhanced the toxicity of spirodiclofen in PSR-TK *P. ulmi* strain, suggesting the involvement of CYPs in spirodiclofen resistance in both tetranychid species. The upregulation of a CYP (*CYP392E10*, *tetur27g01030*) and a CCE (*TuCCE-04*, *tetur01g10750*) was also strongly associated with spirodiclofen resistance in two unrelated *T. urticae* strains (SR-VP and SR-TK). Furthermore, functional expression confirmed that CYP392E10 metabolizes spirodiclofen [20]. Remarkably, the upregulated *P. ulmi* CCE and CYP in the PSR-TK strain cluster in different clades in our phylogenetic analyses of tetranychid CCEs and CYPs than those that are upregulated in the spirodiclofen-resistant *T. urticae* strains (Figs. 2 and 3). Thus, within the Tetranychidae, two different species are able to develop a similar resistance mechanism to spirodiclofen, but the enzymes recruited for this purpose do not need to have followed the same evolutionary path. This independent development of resistance in related species is further reinforced by the fact that only few of the differentially expressed contigs in the PSR-TK *P. ulmi* strain have a BLASTx hit with amino acid translations of differentially expressed genes in two spirodiclofen resistant *T. urticae* strains (SR-VP and SR-TK, [20]) (see Additional file 18). Overall, the analysis has pointed to a number of genes and enzymes that might be causal for the resistant phenotype, and future work including functional expression should confirm that the enzymes can metabolize or sequester spirodiclofen leading to resistance, as was recently undertaken for the model species *T. urticae* [20].

## Conclusions

Using paired-end strand specific reads from RNA of spirodiclofen susceptible and resistant *P. ulmi* strains we generated a *P. ulmi* transcriptome dataset containing 27,777 contigs. As such, our transcriptome data represents a significant increase in the genomic resources that are available for this species. Phylogenetic analyses revealed that in the majority of cases detoxification gene (CCEs, CYPs, UGTs, ABCs) classes/clades/subfamilies are more numerate in *T. urticae* compared to *P. ulmi*, suggestive of a link between detoxification gene proliferation and the polyphagous nature of *T. urticae*. Specific radiations of subfamilies in *P. ulmi* were also observed. Annotation of all major target-sites in *P. ulmi* revealed the presence of mutations in AchE that are likely to confer carbamate and organophosphate resistance. Finally, we used the replicated RNAseq data to assess differences in gene expression between a spirodiclofen resistant and susceptible *P. ulmi* strain and found that a CYP and CCEs are likely to be associated with spirodiclofen resistance in *P. ulmi*. These results are in line with a previous report on molecular mechanisms of spirodiclofen resistance in *T. urticae*. However, the upregulated CYP and CCE in the resistant strains from both species seem not to have evolved from the same common ancestor, indicating both species developed spirodiclofen resistance in a similar but nevertheless independent evolutionary manner. To conclude, the new genomic resources and data that we present in this study for *P. ulmi* will substantially facilitate molecular studies of underlying mechanisms involved in acaricide resistance.

## Methods

### Mite strains

Mites from the three strains (HS, PSR-TK and Ge 16/09) used in this study were all identified morphologically as *P. ulmi* by Johan Witters (ILVO, Belgium) but also on the basis of cytochrome b (cytB) sequence similarity (cytB sequences of the HS and PSR-TK *P. ulmi* strain (Additional file 14) had their best BLASTx hit (E-value < 1E^−100^) with *P. ulmi* cytochrome b (YP_002808558.1) in the NCBI database. The HS strain was originally collected in 1990 from an apple orchard in Burscheid, Germany and is susceptible to most currently used acaricides, including spirodiclofen [17]. The Ge 16/09 is a field strain, collected in 2009 from an orchard in Heidenfahrt-Heidesheim, Germany, and characterised by high resistance levels to mite growth inhibitors hexythiazox and clofentezine (extrapolated resistance ratios in comparison with the susceptible HS strain were 20,000 and 3900 respectively) and moderate resistant to spirodiclofen with a reported spirodiclofen resistance ratio of 59 compared to the HS strain. The PSR-TK strain is a spirodiclofen-selected laboratory strain derived from Ge 16/09 and with a resistance ratio of more than 7000 compared to the HS strain [17]. All mite strains were reared under identical conditions in climatically controlled chambers at 24 +/− 1 °C, 60 % relative humidity and a 16:8 light:dark period for more than 10 generations on domestic plum trees (*Prunus domestica* L. var. Brompton). The PSR-TK strain was maintained under constant selection pressure by rearing on plum trees sprayed with 1000 mg a.i./L spirodiclofen until runoff.

### RNA extraction, library construction, sequencing and assembly of the *P. ulmi* transcriptome

Total RNA was extracted from 200 1–3 day old adult female *P. ulmi* mites from the PSR-TK and HS strains using the RNEasy mini kit (Qiagen, Belgium) with four-fold biological replication (i.e., four replicates each for PSR-TK and HS). Each RNA sample was processed for sequencing by the sequencing service company Fasteris SA (Switzerland) according to the “HiSeq Service Stranded Standard Protocol”. The resulting library was sequenced by Fasteris SA (Switzerland) using the HiSeq 2000 Illumina technology generating strand-specific paired-reads of 2x100 bp. Adapter sequences were removed from the obtained 2x 100 bp reads by Fasteris SA and adapter trimmed reads were used for further analysis. Previously it was shown that a representative transcriptome assembly can be generated from a sub-sample of reads [40]. Hence, to reduce computation time, a subset (the first 12 million paired-reads/sample, 48 million reads/strain, 96 million paired-reads in total) of the total number of reads was used for *P. ulmi* transcriptome assembly using the CLC Genomics Workbench (CLC) software version 6.5.1 and default settings (mapping mode = fast, automatic bubble size = yes, minimum contig length = 200 nt, automatic word size = yes, perform scaffolding = yes, auto-detect paired distances = yes). Using the same subset of reads we also used an alternative approach to assemble the *P. ulmi* transcriptome. Reads were first trimmed using Sickle [79] with quality-cutoff set at 30 and length cutoff set at 90. Trimmed reads were assembled using the Velvet/Oases package [80] and with the following settings for Oases: −-kmin = 59 --kmax = 61 --kstep = 2 --merge = 61 –d “-shortPaired -strand_specific” -p “ins_length = 200”. Transcripts with maximum sequence length (30,044 transcripts) were filtered from the resulting Velvet/Oases assembly (44,903 transcripts) and used for further analysis. To compare the quality of both assemblies all RNA-seq reads (4 replicates/strain) were mapped using Bowtie version 2.1.0 [81] against either the CLC or Velvet/Oases assembly (see Differential expression analysis section below for mapping procedures).

Raw reads have been submitted to the NCBI Short Read Archive (SRA; experiment accession number SRX833872 (HS) and SRX833917 (PSR-TK)). The CLC assembly was submitted to the NCBI Transcriptome Shotgun Assembly (TSA) Sequence Database and deposited at DDBJ/EMBL/GenBank under the accession GCAC00000000. The version described in this paper is the first version, GCAC01000000. *P. ulmi* contigs containing more than 10 % of unassigned nucleotides (N) or more than 14 Ns in a row (1630 sequences) and contigs that were shorter than 200 nt after removal of vector contamination sequences (2 contigs) were not uploaded to the TSA Database following database submission procedures. After uploading, an NCBI contamination screen indicated that 227 out of the 26,145 uploaded sequences should be excluded as they showed very high identity with either rRNA of Bacteria, genes of *Prunus* sp. (host plant of *P. ulmi* strains) or *Wolbachia* sp. sequences. These sequences were removed from the TSA assembly and, finally, 25,918 contigs were uploaded. The complete collection of the CLC assembly (27,777 contigs) was added to this manuscript in Additional file 19. The (alternative) Velvet/Oases *P. ulmi* assembly was added as Additional file 13 to this manuscript.

### Blast homology searches and sequence annotation

All *P. ulmi* contigs (27,777) were used for BLASTx searches against the NCBI non-redundant (nr) protein database (version of September 3^rd^ 2014; E-value cut-off of 1E^−5^) using Standalone BLAST 2.2.30+ [82]. Sequences that did not yield BLASTx hits were subsequently searched against the non-redundant nucleotide database using BLASTn and with an e-value cut-off of 1E^−5^. As *T. urticae* is not yet included in the NCBI nr protein database, *P. ulmi* contigs were also used as query in BLASTx against the *T. urticae* proteome (version March 2014, http://bioinformatics.psb.ugent.be/orcae/overview/Tetur). All BLAST results were imported in Blast2GO (version 2.8) to assign gene ontology (GO) terms to sequences retrieved by BLAST search [83]. Further annotation was done with InterPro, where protein motifs were directly queried at the InterProScan web service and consequently merged to the existing annotation [84]. The annotation results were further fine-tuned with (1) the Annex function in order to augment the annotation through the inference of biological process and cellular component terms from molecular function annotations [85] and with (2) the generic GO slim reduction to summarize the functional information of the transcriptome dataset. Finally, to enable better visualisation of the results, Gene Ontology relationships and enzyme codes (ECs) were highlighted on the Kyoto Encyclopedia of Genes and Genomes (KEGG) maps.

### Analysis of genes related to xenobiotic detoxification

Contigs encoding putative *P. ulmi* CYPs, CCEs, GSTs, UGTs and ABC proteins were retrieved from the *P. ulmi* transcriptome (CLC assembly) by a tBLASTn search and using protein sequences of *T. urticae* CYPs, CCEs, GSTs, UGTs and ABCs as query [21, 39, 57]. tBLASTn searches were performed with NCBI Standalone BLAST 2.2.30+ [82] and a cut-off E-value < 1E^−5^. Open reading frames (ORFs) of *P. ulmi* contigs encoding detoxification enzymes and ABC proteins were identified using “EMBOSS 6.3.1 getorf” integrated in the Mobyle portal framework (http://mobyle.pasteur.fr/). *P. ulmi* ORFs were aligned against each other using BLASTn. In cases where two ORFs showed more than 94 % identity at the nucleotide level, they were considered as allelic variants and the longest ORF was retained for phylogenetic analysis. ORFs with identical overlapping sequences were considered to be part of the same gene and merged using BioEdit version [86], except if the merged ORF misaligned when blasted to the *T. urticae* protein database.

Two previously published *P. citri* transcriptomes were also mined for transcripts encoding detoxification enzymes and ABC transporters. For both published *P. citri* transcriptomes, the cDNA library was constructed using a mixture of RNA extracted from different developmental stages and subsequently sequenced using Illumina HiSeq 2000 technology with a read-length of 90 bp [33, 34]. However, as the *P. citri* transcriptome sequence assemblies (TSAs) have not been made publicly available, we assembled these transcriptomes *de novo* using a similar approach as for *P. ulmi* and using all reads in the sequence read archives (SRAs) ERR044692-ERR044695 and SRR341928 provided by the studies of Liu et al. [33] and Niu et al. [34], respectively. *P. citri* ORFs encoding CYPs, CCEs, GSTs, UGTs, and ABC proteins were identified using a similar approach as for *P. ulmi*. Likewise, these *P. citri* ORFs were aligned against each other using BLASTn. In case two *P. citri* contigs showed at least 94 % identity they were considered as allelic variant and the longest ORF was retained for phylogenetic analysis.

A final selection of *Panonychus* ORFs encoding CYPs, GSTs UGTs and ABCs was translated into protein sequences and aligned with their homologues in *T. urticae* using MUSCLE version 3.8.31 [87]. Alignments were inspected by eye and for each alignment only protein sequences showing no misalignment and having a minimum ORF length (CYPs: 450 nt, CCEs: 450 nt, GSTs: 300 nt, UGTs: 450 nt) were retained for the final alignment. Two phylogenetic analyses were performed for the CYP gene family: one with all tetranychid CYPs and one restricted to tetranychid and *D. melanogaster* mitochondrial CYPs (see [88]). *Panonychus* mt CYPs were identified based on the phylogenetic analysis of all tetranychid CYPs. For the phylogenetic analysis of CCEs, a reference set of arthropod CCE protein sequences was also included in the alignment (Additional file 9). As N- and C termini of CCEs are highly variable, the alignment of CCE protein sequences was trimmed as previously described [49]. Only the nucleotide binding domain (NBD) of ABC proteins encoded by *Panonychu*s ORFs was used for phylogenetic analysis of ABC proteins. NBDs were extracted using ScanProsite (http://prosite.expasy.org/scanprosite/) and the PROSITE profile PS5089. Next to *T. urticae* ABC transporter NBDs, C- and N terminal NBDs of *D. melanogaster* ABC transporters were also included in phylogenetic analysis of tetranychid ABC transporter NBDs [57]. Model selection was done with ProtTest 2.4 and according to the Akaike information criterion WAG + G + F, LG + I + G + F, LG + I + G + F, LG + I + G, WAG + G + F and LG + G + F were optimal for the phylogenetic reconstruction of CYPs, mitochondrial CYPs, UGTs, GSTs, CCEs and ABC proteins, respectively. Finally, for each alignment a maximum likelihood analysis was performed using Treefinder (version 2011) [89] bootstrapping with 1000 pseudoreplicates (LR-ELW). The resulting trees were midpoint rooted and edited with MEGA 6.0 software [90].

### Annotation of horizontally transferred genes (excluding UGTs)

Annotation of *P. ulmi* and *P. citri* contigs encoding horizontally transferred genes (HTGs) was done in a similar way as for genes involved in xenobiotic detoxification. Model selection and phylogenetic analysis of IDRCD genes was done in a similar way as for genes involved in xenobiotic detoxification. According to the Akaike information criterion WAG + I + G + F was optimal for phylogenetic reconstruction of tetranychid IDRCDs.

### Identification of *P. ulmi* target-site sequences

To obtain *P. ulmi* target site sequences as full length as possible we mined both *P. ulmi* transcriptomes (CLC and Velvet/Oases, see above) for contigs encoding target-sites of acaricides using tBLASTn (E-value cutoff < 1E^−5^) and *T. urticae* target-site protein sequences as query. *P. ulmi* contigs encoding target-sites (from both transcriptomes (CLC and Velvet/Oases) were aligned against each other and those contigs having identical overlapping regions were merged. The resulting contigs were manually assembled to obtain *P. ulmi* target-site sequences as full length as possible. All reads were then mapped against the *P. ulmi* target site sequences as for the differential expression analysis (see below) with this difference that resulting BAM files were merged for each strain using the SAMtools package [91]. For both the spirodiclofen susceptible and the resistant *P. ulmi* strain, a consensus sequence was then derived using a perl script (gene_extractor.pl) written by Rutger Vos and available at https://github.com/naturalis/fastq-simple-tools/tree/master/script. The obtained *P. ulmi* target site sequences were screened for all target site mutations previously described in the literature [42].

### Differential expression analysis

To assess differential gene expression between the HS and PSR-TK strains, we first took the reverse complement of each *P.ulmi* contig where the majority of its BLASTx hits mapped on the reverse strand. *P. ulmi* contigs were subsequently concatenated into a single sequence with spacers (“Ns”) of length 100 added between contigs using a custom python script. For each RNA-seq replicate (4 per strain, 8 in total), reads were mapped against this concatenated sequence using Bowtie version 2.1.0 [81] with the preset option “--very-sensitive-local” and with the maximum fragment length for valid paired-end alignments set to 1000 (−X 1000). Mapped reads were counted using htseq-count that is included in the HTSeq package [92]. Forward strand reads were counted using the “--stranded = reverse” option while the “--stranded = yes” option was used to count reverse strand reads. Those *P. ulmi* contigs of which the proportion between the number of reads mapping to one strand and the total number of reads mapping to either the forward or the reverse strand was more than or equal to 0.95 were considered to have strand-specific reads (20,069 contigs, 72,3 % of all contigs). Read counts of *P. ulmi* contigs with (1) a BLAST hit, (2) strand-specific reads and (3) not considered contamination (see above) were used for differential expression analysis. Differentially expressed contigs between the resistant (PSR-TK) and susceptible (HS) *P. ulmi* strain were determined using the DESeq2 (version 1.6.3;[92]) and Bioconductor (http://bioconductor.org/) R-packages. The “unfiltered DESeq2 results” settings (dds < − DESeq(dds, minReplicatesForReplace = Inf) and res < − results(dds, cooksCutoff = FALSE, independent Filtering = FALSE)) were used for differential expression analysis. *P. ulmi* contigs with a fold change higher than two and a Benjamini-Hochberg adjusted *p*-value less than or equal to 0.05 were considered differentially expressed.

### RT-qPCR

A set of eight differentially expressed contigs were evaluated by RT-qPCR. This set included 3 down- and 5 upregulated *P. ulmi* contigs. *P. ulmi* contig_01010 and contig_00741 showed best BLASTx hits with *T. urticae* RP49 (*tetur18g03590*) and ubiquitin (*tetur06g00900*), E-value of 4E^−70^ and 5E^−59^, respectively, and were used as reference genes for RT-qPCR. Primers were designed using Primer3 v.0.4.0 [93] and are listed in Additional file 20. RNA was extracted in triplicate from each strain as described above and 2 μg was reverse transcribed using the Maxima First Strand cDNA synthesis kit for RT-qPCR (Fermentas Life Sciences). All qPCR reactions were conducted with a thermal cycler Mx3005P (Stratagene). Reactions were prepared with Maxima SYBR Green qPCR/Master Mix following the manufacturer’s instructions (Fermentas Life Sciences) and run in two technical and three biological replicates. Non-template-controls were also included to eliminate potential contamination of the samples. The qPCR run protocol was as follows: initial denaturation at 95 °C for 10s, 35 cycles of 95 °C for 15 s, 55 °C for 30s, 72 °C for 30s. To exclude the possibility of nonspecific amplification, each PCR was followed by a melting curve step (ramping from 95 to 55 °C by 1 °C every 2 s) to confirm a single amplicon. Amplification efficiency of each primer pair was calculated from the standard curve, prepared of cDNA of all samples tested, with a dilution range from 0.4 to 50 ng RNA. The Ct values for *P. ulmi* contig_01010 (RP49) and contig_00741 (ubiquitin) were used for normalization. Significant differences in the relative expression values of the target genes were tested with pairwise fixed reallocation randomization [94, 95].

## Additional files

Additional file 1: Table S1. Alignment summary for the CLC and Velvet/Oases assemblies. (XLSX 16 kb) Additional file 2: File S1. Complete transcriptome assembly of *P. citri* reassembled from raw reads data [33]. (Format: FASTA, examination of this file requires the BioEdit program available at http://www.mbio.ncsu.edu/bioedit/bioedit.html. (FAS 19163 kb) Additional file 3: File S2. Complete transcriptome assembly of *P. citri* reassembled from raw reads data [34]. (Format: FASTA, examination of this file requires the BioEdit program available at http://www.mbio.ncsu.edu/bioedit/bioedit.html). (FAS 21895 kb) Additional file 4: Table S2. BLAST2GO analysis of *P. ulmi transcriptome*. (XLSX 12041 kb) Additional file 5: Figure S1. Taxonomic distribution of top BLAST hits of the *P. ulmi* transcriptome. Analysis of the taxonomic distribution of the 11,673 top BLAST hits obtained by BLASTx and BLASTn against the non-redundant protein and nucleotide database (NCBI) respectively, and by BLASTx against the *T. urticae* proteome (http://bioinformatics.psb.ugent.be/orcae/overview/Tetur). Wheel diagram depicts the percentage distribution within different taxonomic groups. (PDF 25 kb) Additional file 6: Figure S2. Enzyme Classification (EC) analysis of the *P. ulmi* transcriptome. The wheel diagram depicts percentage distribution of EC numbers in general EC terms. (PDF 26 kb) Additional file 7: Table S3. Annotation details and accession numbers of Cytochrome P450s. (XLSX 154 kb) Additional file 8: Figure S3. Phylogenetic analysis of *P. ulmi* mitochondrial CYPs. Maximum likelihood phylogenetic tree of *P. ulmi*, *P. citri*, *T. urticae* and *D. melanogaster* mitochondrial CYP protein sequences. The tree was rooted with *T. urticae* CYP307A1, *P. ulmi* PcP450_42 and *P. citri* Pc0cP450_21 (CYP2 clan members). The scale bar represents 0.5 amino-acid substitutions per site. Numbers at the branch point of each node represent the bootstrap value resulting from 1000 pseudoreplicates (LR-ELW). Colour and shape codes are as follows: *P. ulmi,* green triangle, *P. citri,* yellow triangle, *T. urticae,* red square and *D. melanogaster,* black dot. (PDF 1.35 MB) Additional file 9: Table S4. Annotation details and accession numbers of the carboxylcholinesterases. (XLSX 171 kb) Additional file 10: Table S5. Annotation details and accession numbers of Glutathione S- transferases. (XLSX 57 kb) Additional file 11: Table S6. Annotation details and accession numbers of ATP-binding cassette family transporters. (XLSX 441 kb) Additional file 12: Table S7. Annotation details and accession numbers of HTGs. (XLSX 159 kb) Additional file 13: File S3. Alternative transcriptome assembly (Velvet/Oases) of *P. ulmi* used for obtaining target-site sequences as full length as possible and as an extra resource for mining of HTGs. (Format: FASTA, examination of this file requires the BioEdit program available at http://www.mbio.ncsu.edu/bioedit/bioedit.html. (FAS 20474 kb) Additional file 14: File S4. Target site nucleotide sequences of the *P. ulmi* PSR-TK and HS-strain. (Format: FASTA, examination of this file requires the BioEdit program available at http://www.mbio.ncsu.edu/bioedit/bioedit.html). (FAS 67 kb) Additional file 15: Figure S4. ClustalW alignment of ACCase protein sequences of the PSR-TK and HS *P. ulmi* strain and *T. urticae.* The carboxyltransferase domain is shaded in grey while the residue corresponding to the E645K substitution associated with spiromesifen resistance in *T. vaporarorium* is shaded in yellow. (DOC 261 kb) Additional file 16: Table S8. The number of mapped RNA reads per contig for both *P. ulmi* strains (HS and PSR-TK). Mapped reads were counted using htseq-count, included in the HTSeq python package [92]. (XLSX 2544 kb) Additional file 17: Table S9. Differentially expressed contigs in the *P. ulmi* PSR-TK strain relative to the HS strain (Benjamini-Hochberg adjusted *p*-value ≤ 0.05 and │FC│ > 2). (XLSX 30 kb) Additional file 18: Table S10. Differentially expressed contigs in the *P. ulmi* PSR-TK strain with a BLASTx (E-value treshold e-15) hit against amino acid translations of differentially expressed genes in SR-VP and SR-TK, two spirodiclofen resistant *T. urticae* strains [20]. (XLSX 48 kb) Additional file 19: File S5. Transcriptome assembly (CLC Genomics Workbench) of *P. ulmi* described in this study. (Format: FASTA, examination of this file requires the BioEdit program available at http://www.mbio.ncsu.edu/bioedit/bioedit.html). (FAS 28273 kb) Additional file 20: Table S11. qPCR primers used in this study. Sequences are given for the forward (F) and reverse primer (R). (DOCX 13 kb)
