# Supplementary figures and images for: Transcriptome profiling of a spirodiclofen susceptible and resistant strain of the European red mite Panonychus ulmi using strand-specific RNA-seq
Source: BMC Genomics. 2015 Nov 18;16:974. doi: 10.1186/s12864-015-2157-1 (PMC4652392; doi:10.1186/s12864-015-2157-1)

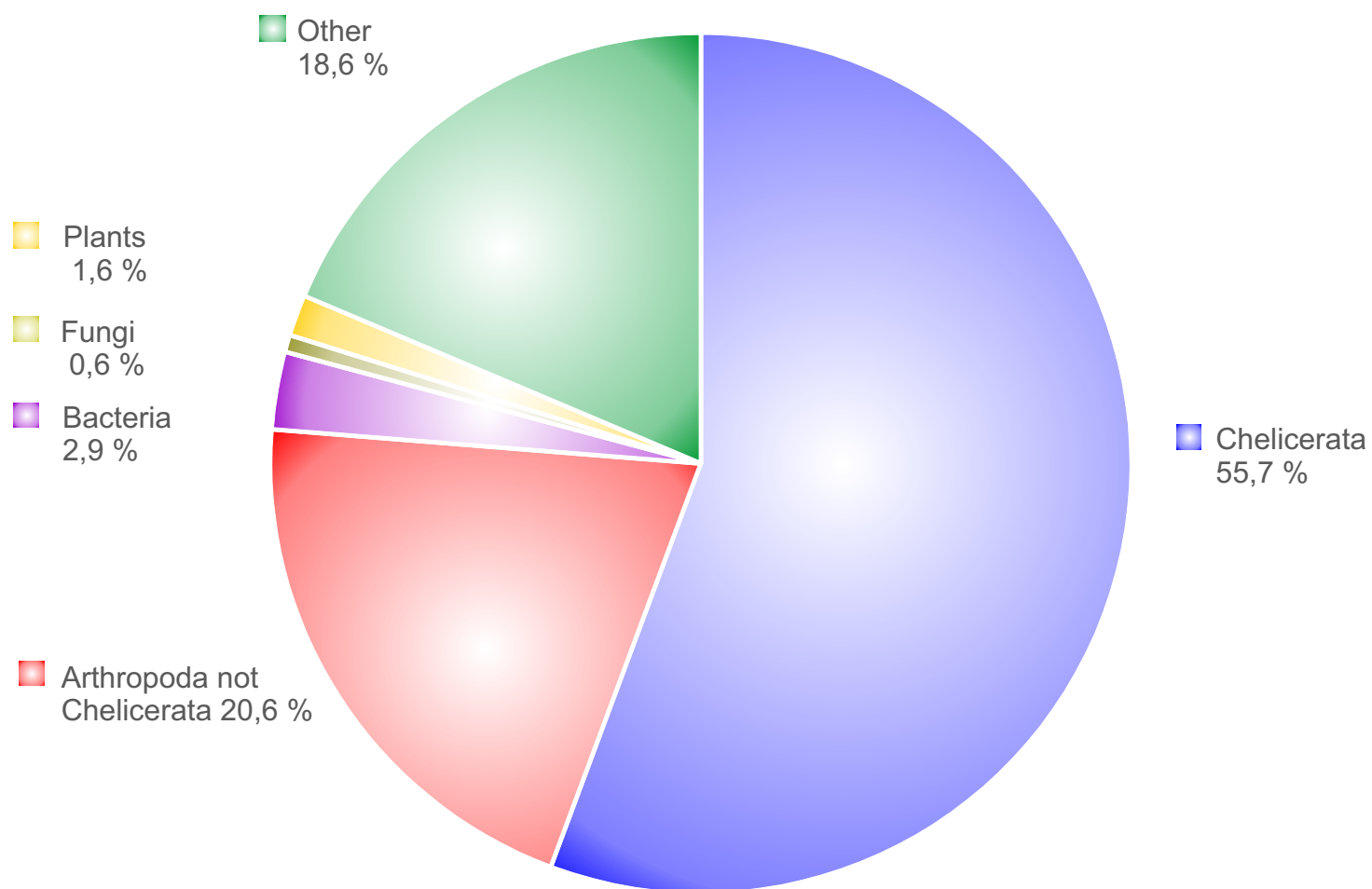

Supplement: Additional file 5: Figure S1. — Taxonomic distribution of top BLAST hits of the P. ulmi transcriptome. Analysis of the taxonomic distribution of the 11,673 top BLAST hits obtained by BLASTx and BLASTn against the non-redundant protein and nucleotide database (NCBI) respectively, and by BLASTx against the T. urticae proteome (http://bioinformatics.psb.ugent.be/orcae/overview/Tetur). Wheel diagram depicts the percentage distribution within different taxonomic groups. (PDF 25 kb) [file 12864_2015_2157_MOESM5_ESM.pdf]

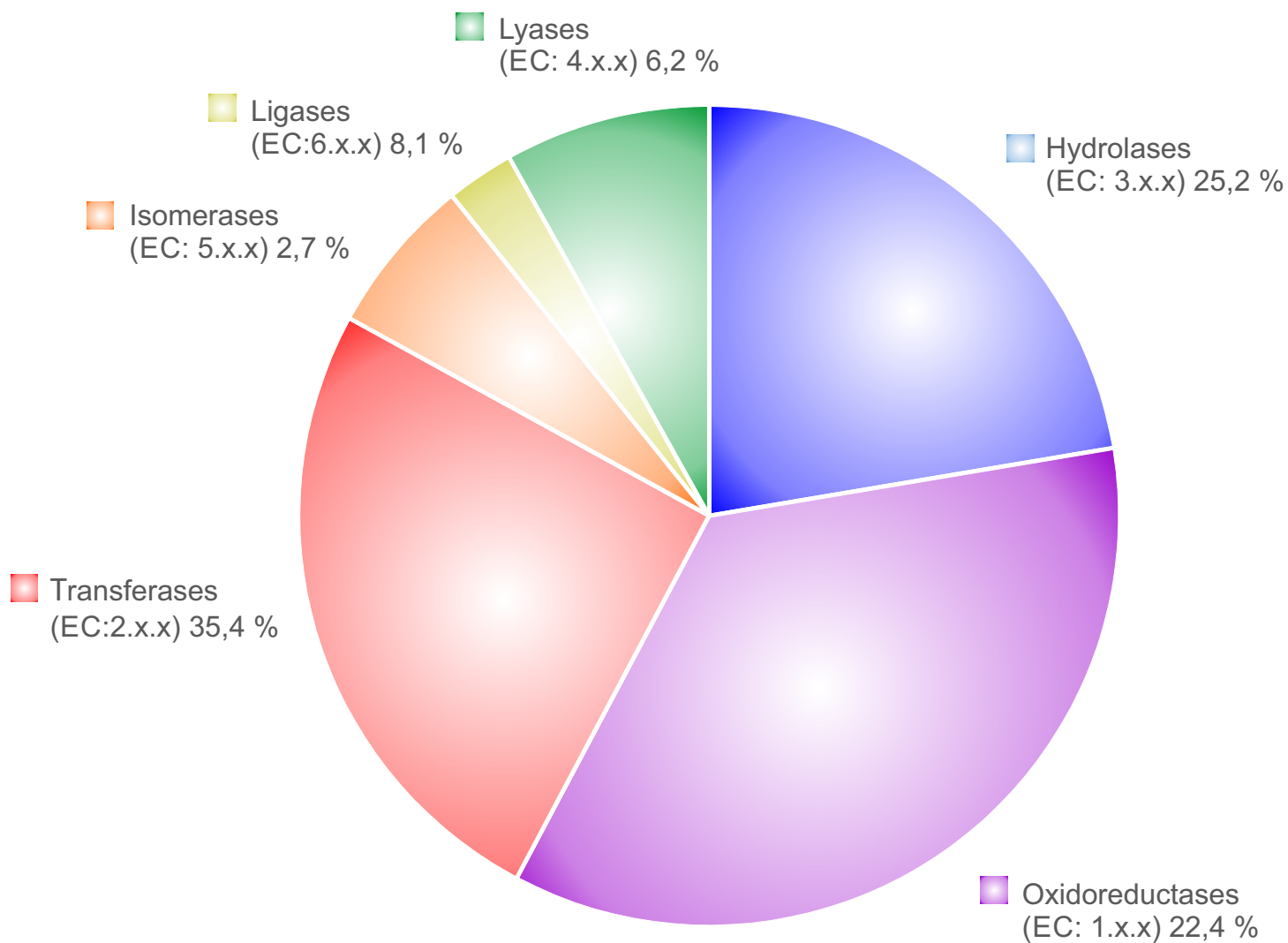

Supplement: Additional file 6: Figure S2. — Enzyme Classification (EC) analysis of the P. ulmi transcriptome. The wheel diagram depicts percentage distribution of EC numbers in general EC terms. (PDF 26 kb) [file 12864_2015_2157_MOESM6_ESM.pdf]

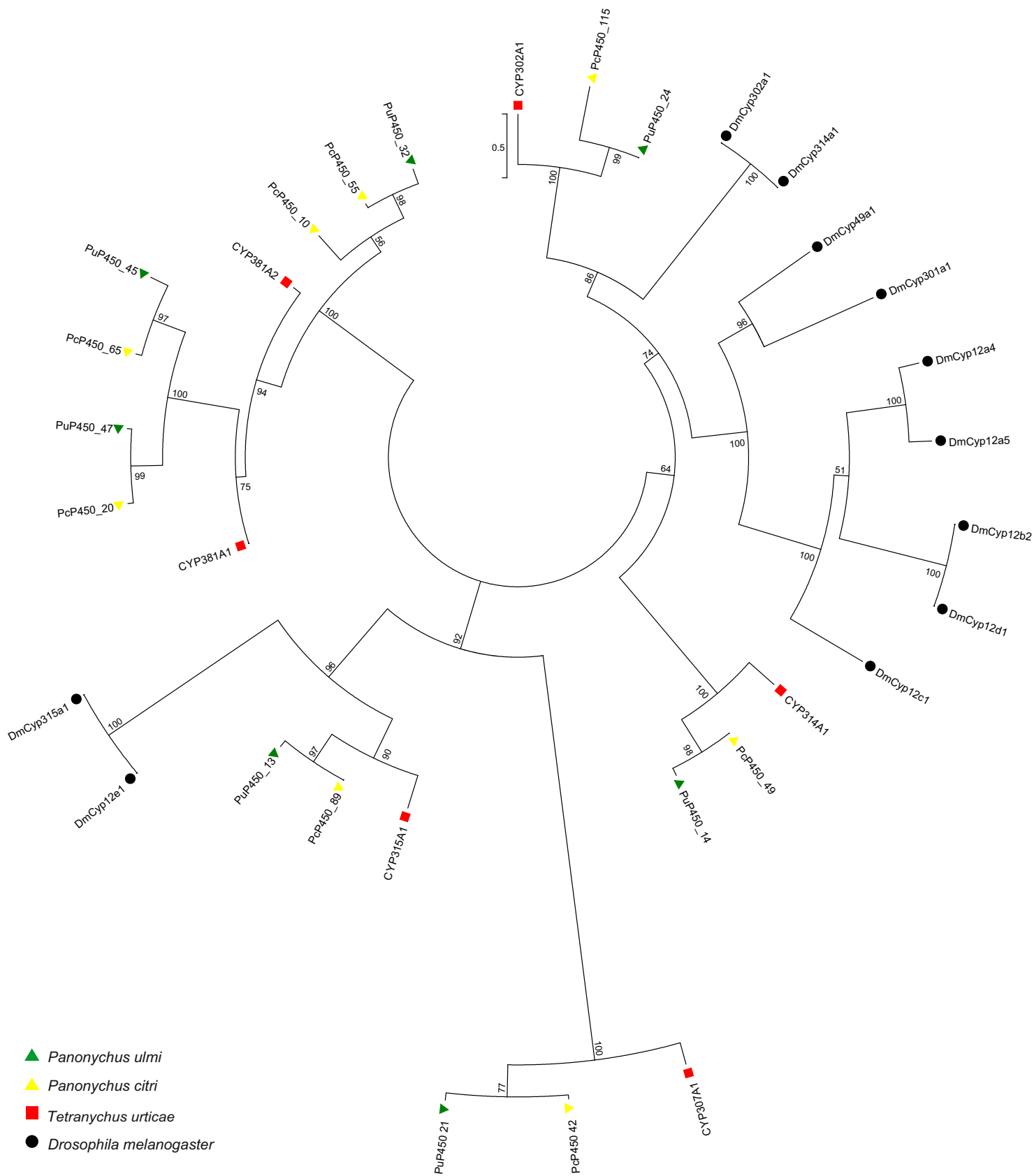

Supplement: Additional file 8: Figure S3. — Phylogenetic analysis of P. ulmi mitochondrial CYPs. Maximum likelihood phylogenetic tree of P. ulmi, P. citri, T. urticae and D. melanogaster mitochondrial CYP protein sequences. The tree was rooted with T. urticae CYP307A1, P. ulmi PcP450_42 and P. citri Pc0cP450_21 (CYP2 clan members). The scale bar represents 0.5 amino-acid substitutions per site. Numbers at the branch point of each node represent the bootstrap value resulting from 1000 pseudoreplicates (LR-ELW). Colour and shape codes are as follows: P. ulmi, green triangle, P. citri, yellow triangle, T. urticae, red square and D. melanogaster, black dot. (PDF 1.35 MB) [file 12864_2015_2157_MOESM8_ESM.pdf]
