# Supplementary material for: Transcriptome profiling of a spirodiclofen susceptible and resistant strain of the European red mite Panonychus ulmi using strand-specific RNA-seq
Source: BMC Genomics. 2015 Nov 18;16:974. doi: 10.1186/s12864-015-2157-1 (PMC4652392; doi:10.1186/s12864-015-2157-1)
Supplement: Additional file 15: Figure S4. — ClustalW alignment of ACCase protein sequences of the PSR-TK and HS P. ulmi strain and T. urticae. The carboxyltransferase domain is shaded in grey while the residue corresponding to the E645K substitution associated with spiromesifen resistance in T. vaporarorium is shaded in yellow. (DOC 261 kb) [file 12864_2015_2157_MOESM15_ESM.doc]

10 20 30 40 50 60 70 80 90 100

....|....|....|....|....|....|....|....|....|....|....|....|....|....|....|....|....|....|....|....|

**Pulmi_ACCAse_HS**  **--GPNLCSHEVDA--IPTPEEFVNRFDGLKVINKVLIANNGIAAVKX-----------------IRFIVMVTPEDLKANAEYIKMADHYVPVPGGPNNNN**

**Pulmi_ACCase_PSR-TK** **--...........--...........Y....................-----------------..................V.................**

**tetur21g02170**  **MS....S.QDQ..AI.S......R....R...KT............CMRSIRRWAYEIFRNERA...V................................**

110 120 130 140 150 160 170 180 190 200

....|....|....|....|....|....|....|....|....|....|....|....|....|....|....|....|....|....|....|....|

**Pulmi_ACCAse_HS**  **YANVDLIVDIAKRTKAQGVWAGWGHASENPKLPELLTKAGIAFMGPPHNAMWALGDKIASSIVAQTAKVPTLSWSGSGLVASNYVEGKPIKISSDLYRKG**

**Pulmi_ACCase_PSR-TK** **...............S..............R.........................................A...............T...........**

**tetur21g02170**  **...........I.M..............Y.R.....N...........H..................R............D............A......**

210 220 230 240 250 260 270 280 290 300

....|....|....|....|....|....|....|....|....|....|....|....|....|....|....|....|....|....|....|....|

**Pulmi_ACCAse_HS**  **CVTDEDEGLEAAYKIGFPVMIX------------------------------------------------------------------------------**

**Pulmi_ACCase_PSR-TK** **......................------------------------------------------------------------------------------**

**tetur21g02170**  **..S..V.....S.........KASEGGGGKGIRKCESADEFSIKFRQVQNEVPGSPIFIMKLATCARHLEVQLLADQYGNAISLFGRDCSIQRRHQKIIE**

310 320 330 340 350 360 370 380 390 400

....|....|....|....|....|....|....|....|....|....|....|....|....|....|....|....|....|....|....|....|

**Pulmi_ACCAse_HS**  **------AIEETFAEMEDAAVRLAKMVGYESAGTVEYLYDPAEKKFYFLELNPRLQVEHPCSEMVSDVNLPACQLQIAMGLPLYRIKDIRLLYGESPLVDK**

**Pulmi_ACCase_PSR-TK** **------..............................................................................................**

**tetur21g02170**  **EAPCVI.N..A.....E.............T...........N...................................................P..N.N**

410 420 430 440 450 460 470 480 490 500

....|....|....|....|....|....|....|....|....|....|....|....|....|....|....|....|....|....|....|....|

**Pulmi_ACCAse_HS**  **PIDFRVGRAYPRGHVIAARITSENPDEGFKPSSGTVQELNFRSNKNVWGYFSVGASGGLHEYADSQFGHCFSWGEDREEARENLVIALKELSIRGDFRTT**

**Pulmi_ACCase_PSR-TK** **.....................................................S..............................................**

**tetur21g02170**  **....KL...Q.W.................................................F................Q.....................**

510 520 530 540 550 560 570 580 590 600

....|....|....|....|....|....|....|....|....|....|....|....|....|....|....|....|....|....|....|....|

**Pulmi_ACCAse_HS**  **VEYLITLLETEAFQRNEIDTGWLDKLISERVQSDKPKTTIALICGSIHVADTKFSANWQNFESSLQKGQIPHSNTLNNFISVELIYENVKYLVYVAKSGP**

**Pulmi_ACCase_PSR-TK** **...............................................................X....................................**

**tetur21g02170**  **..............K.......................SL...........AQ..G................AT..T.SVN...........FN......**

610 620 630 640 650 660 670 680 690 700

....|....|....|....|....|....|....|....|....|....|....|....|....|....|....|....|....|....|....|....|

**Pulmi_ACCAse_HS**  **NSYFLVLNDSFKEVEFHQMSDSGLLLSVDGSSHVTYMKEEIDQYRVIVANQTCVFSKENDPTILRSPSPGKLQQFLVEDGSHVSSGQPYAEIEVMKMIMT**

**Pulmi_ACCase_PSR-TK** **....................................................................................................**

**tetur21g02170**  **....V.....Y....V................I...................................................A...............**

710 720 730 740 750 760 770 780 790 800

....|....|....|....|....|....|....|....|....|....|....|....|....|....|....|....|....|....|....|....|

**Pulmi_ACCAse_HS**  **LTTQESGIIRHAXRFGAVLEAGCILARLDLDDPTKVRRAELYTEGWPELAYRDTGGKVATEADSHHPQKLNVIFSSVKSHLENYLAGYVLPEPFFTNKMQ**

**Pulmi_ACCase_PSR-TK** **............K.................................................................................Y.....**

**tetur21g02170**  **...S........K...S.........................S...Q..MA..IV..NPS.T..A.......T..........F..........Y.T...**

810 820 830 840 850 860 870 880 890 900

....|....|....|....|....|....|....|....|....|....|....|....|....|....|....|....|....|....|....|....|

**Pulmi_ACCAse_HS**  **ENVTXFMKXLKDPQLPLLELQDIISSKSGRIPPTVESSIKKLMQKYGKNITAVLQIFPSQEIALVIDSYAATLEKRSDRDVFFLNTQGIVQLVQRYRNGI**

**Pulmi_ACCase_PSR-TK** **....................................................................................................**

**tetur21g02170**  **....L..RC.................Q......S............A................S....................................**

910 920 930 940 950 960 970 980 990 1000

....|....|....|....|....|....|....|....|....|....|....|....|....|....|....|....|....|....|....|....|

**Pulmi_ACCAse_HS**  **RGRLRSCVQDLLKNYIDVEQHFQAGDYDKCVSTLRDKYVDEGMSKAVANIFSHLSVAKKNQLIIKLIDHLARQEPGLTEELSNILNALTALNRSENVKVA**

**Pulmi_ACCase_PSR-TK** **....................................................................................................**

**tetur21g02170**  **................................A.TE..K...............N................G............................**

1010 1020 1030 1040 1050 1060 1070 1080 1090 1100

....|....|....|....|....|....|....|....|....|....|....|....|....|....|....|....|....|....|....|....|

**Pulmi_ACCAse_HS**  **LRARQVLITAHQPPYALRFSQMEAMFLSVIDMYSHEFDANNMNKLIISETSIFDVLHQFFYHSNIVVRKASLEVYVRRAYICYEMVALQHLFIRNCSKGL**

**Pulmi_ACCase_PSR-TK** **....................................................................................................**

**tetur21g02170**  **........S......................................................................................SPN-D**

1110 1120 1130 1140 1150 1160 1170 1180 1190 1200

....|....|....|....|....|....|....|....|....|....|....|....|....|....|....|....|....|....|....|....|

**Pulmi_ACCAse_HS**  **STQTELANCYTENNDSVIPTAVFHFILPSSHPSISSQPLAATVEAYSNSYEESSNGQLHSNYRIGVIAAFKNLEHVEEHFDSLLNACTEYENDLSDEX--**

**Pulmi_ACCase_PSR-TK** **............................................D.....................................................--**

**tetur21g02170**  **.GSI..S.S.A.Q.....................A...QSQNT.GL..NFSGE.SAH.............................L.DFDK..AA.HFD**

1210 1220 1230 1240 1250 1260 1270 1280 1290 1300

....|....|....|....|....|....|....|....|....|....|....|....|....|....|....|....|....|....|....|....|

**Pulmi_ACCAse_HS**  **------SSSPTNDVSSSDMNDFNGSDKTMTNDDNDNESKAEPIHIINIAIKPEGADDENLSKKCESFCQTRSKXLIEQGVRRVTFIVCSLYCFPRYFTYR**

**Pulmi_ACCase_PSR-TK** **------.................................................................N.Q..........................**

**tetur21g02170**  **APESNHQ..SPS.KG.....GY..P.----TEESQ.KESN..................I......A...S...L...R..........N...........**

1310 1320 1330 1340 1350 1360 1370 1380 1390 1400

....|....|....|....|....|....|....|....|....|....|....|....|....|....|....|....|....|....|....|....|

**Pulmi_ACCAse_HS**  **QRDGFVEDRIYRHLEPALAFQLEINRLRNYDLEAIPTANQRMHLYLGKAKSIHTKQLSDYRFFIRTIIRHSDLITKEASYEFLQNEGERFLLEALDELEV**

**Pulmi_ACCase_PSR-TK** **.....................................................A..............................................**

**tetur21g02170**  **.....T.............................................T.....I..........................................**

1410 1420 1430 1440 1450 1460 1470 1480 1490 1500

....|....|....|....|....|....|....|....|....|....|....|....|....|....|....|....|....|....|....|....|

**Pulmi_ACCAse_HS**  **AFTHPSAKQTDCNHIFLNFVPKVTMDPLRIAENVKNTVKNYASRLRKLRVLQAELRMRIRMTTNGKCTPFRLYVSYESGYSLAIHLYKETRDPTSGLTKF**

**Pulmi_ACCase_PSR-TK** **..............................T.....................................................................**

**tetur21g02170**  **.....A.......................................................................................A......**

1510 1520 1530 1540 1550 1560 1570 1580 1590 1600

....|....|....|....|....|....|....|....|....|....|....|....|....|....|....|....|....|....|....|....|

**Pulmi_ACCAse_HS**  **EAWGAPYYEPGPLNDHAISXPYRTKDYLQSKRFLAQSNGTTYIYDFPVMFQTALTQIWREYLKSKEDYEVEIPRNVISYVELVVDFATEKVSEVKRQPGE**

**Pulmi_ACCase_PSR-TK** **...................T................................................................................**

**tetur21g02170**  **...............QP..S.................................................L.M.KT.L.........S.G.MT.QN.....**

1610 1620 1630 1640 1650 1660 1670 1680 1690 1700

....|....|....|....|....|....|....|....|....|....|....|....|....|....|....|....|....|....|....|....|

**Pulmi_ACCAse_HS**  **NECGMVAWRFTIYTPEYPEGRDIIVIGNDITHQLGIFGPQEDIVFNLASVRARELGIPRIYISANSGAKIGLAEEVKSIFQICWIDNNIPDKGFHYLYLS**

**Pulmi_ACCase_PSR-TK** **....................................................................................................**

**tetur21g02170**  **........H..LF..........................R...A....................................E.....K.............**

1710 1720 1730 1740 1750 1760 1770 1780 1790 1800

....|....|....|....|....|....|....|....|....|....|....|....|....|....|....|....|....|....|....|....|

**Pulmi_ACCAse_HS**  **PENYKKVEKSVNAELIEDEGEQRYKILDIVGKESGLGVENXKYAGLIAGETSRAYQDIITITMVTCRAIGIGSYLARLSQRIVQLESSHIILTGHASLNK**

**Pulmi_ACCase_PSR-TK** **........................................L...........................................................**

**tetur21g02170**  **......I...I......................E......L...............EV..........................................**

1810 1820 1830 1840 1850 1860 1870 1880 1890 1900

....|....|....|....|....|....|....|....|....|....|....|....|....|....|....|....|....|....|....|....|

**Pulmi_ACCAse_HS**  **VLGTEVYSSNNQLGGTQIMFANGVSHAKAKDDLEAILVILDWLSYAPKRREKPPQLPILFKAKPSPLPIMEPIDIIDRDVVYCPTSSPYDPRWILNGRES**

**Pulmi_ACCase_PSR-TK** **.......................................................................S.......................S....**

**tetur21g02170**  **................................................K...ATK.VN.T.I..A.......T...........................**

1910 1920 1930 1940 1950 1960 1970 1980 1990 2000

....|....|....|....|....|....|....|....|....|....|....|....|....|....|....|....|....|....|....|....|

**Pulmi_ACCAse_HS**  **QQGVWEEGFFDKGTFHEIMAGWAKTVVCGRARLGGIPLGVVSVETRKTEVIIPADPANPTSETKVLSQAGQVWFPDSAFX--------------------**

**Pulmi_ACCase_PSR-TK** **..............................................T.................................--------------------**

**tetur21g02170**  **P............S.............V.........V..I...V....................Q.............KTAQAINDFNREGLPLMIFAN**

2010 2020 2030 2040 2050 2060 2070 2080 2090 2100

....|....|....|....|....|....|....|....|....|....|....|....|....|....|....|....|....|....|....|....|

**Pulmi_ACCAse_HS**  **WRGFSGGMKDMYDQILKFGALIVDALVAYTQPIIVYIPPYAELRGGAWAVVDTTINPRHMEMYADPDSRGGVIEPEGSVEIRFRIKDQIKAMHRNDIICK**

**Pulmi_ACCase_PSR-TK** **....................................................S...............................................**

**tetur21g02170**  **....................Q........N..................................................................V...**

2110 2120 2130 2140 2150 2160 2170 2180 2190 2200

....|....|....|....|....|....|....|....|....|....|....|....|....|....|....|....|....|....|....|....|

**Pulmi_ACCAse_HS**  **KMIXEINKATDASVKKKLERQLNDREQQIKGAYHIVATYFADLHDKPYNMLMRKVIRGIIPWHRARYYFYWRLRRLSVEEILKCDLESIDVTMTIEQITE**

**Pulmi_ACCase_PSR-TK** **...A................................................................................X...............**

**tetur21g02170**  **.I.A....T..PA.........................................A.........S.........................GS......NN**

2210 2220 2230 2240 2250 2260 2270 2280 2290 2300

....|....|....|....|....|....|....|....|....|....|....|....|....|....|....|....|....|....|....|....|

**Pulmi_ACCAse_HS**  **LMEQWFYADQKNTKTQLWNDDKFVAEWLQMQIDDPSSLVNEKLKELRLNQFSLVIESVCEKFLPEGLNFDDTKKVANCLKTLTDQFSSKAGINCNNINNQ**

**Pulmi_ACCase_PSR-TK** **...............................................................X....................................**

**tetur21g02170**  **...K.........Q.................L...A.....N.......N..Q.V.....Q...K..TSE.SLR..D..R....KLKT.V-VFS.KHSDI**

2310 2320 2330

....|....|....|....|....|....|

**Pulmi_ACCAse_HS**  **LVEGDNSTSKTDSSKNESTSNGVGGDDSSK**

**Pulmi_ACCase_PSR-TK** **...........................X..**

**tetur21g02170**  **EK.AT.-----.GQSV........-NG...**
