# Supplementary material for: Transcriptome profiling of a spirodiclofen susceptible and resistant strain of the European red mite Panonychus ulmi using strand-specific RNA-seq
Source: BMC Genomics. 2015 Nov 18;16:974. doi: 10.1186/s12864-015-2157-1 (PMC4652392; doi:10.1186/s12864-015-2157-1)
Supplement: Additional file 20: Table S11. — qPCR primers used in this study. Sequences are given for the forward (F) and reverse primer (R). (DOCX 13 kb) [file 12864_2015_2157_MOESM20_ESM.docx]

**Additional file 20 - qPCR primers used in this study**

| *P. ulmi* Gene/Contig | Forward (5’-3’) | Reverse (5’-3’) |
| --- | --- | --- |
| RP 49 | GGAAAGCAATTGTTGAACGATCC | CTTCACTCCTGAGTCTAGAGTTGG |
| Ubiquitin | CCAGCAACGTCTCATCTTCAG | CTAAGTGAAGAACCGATCCACC |
| Contig_10595 | ATGAACCTCTGAGTTCCGGG | ACCAAGAGTCCGGTGTTACC |
| Contig_01232 | ACGAATCGAAGCTCTACCTGA | AACCGAATCGAGCCATAGGT |
| Contig_00445 | CCAATTGGCCACCTCATTGT | TCTCCATGTTCAAGAGCCGA |
| Contig_01016 | GAGGAGATTGTTTCATCTTCGGT | GCAAGTCGATAACAAGCCCA |
| Contig_15408 | TCGTTATCTGGGCCATGAGA | GACATCTCCAGTGTGAAGATGT |
| Contig_00577 | TCCGATCACCCGAAAATC | TGCGGTTACATTGAAAGGTG |
| Contig_05939 | TCCTTAAGTCTATGGCGGCC | AAGAGTTGCCCATGAAAGCG |
| Contig_19626 | TTAATAGGATTGTCTTCATGAGTGG | TCGTTTGATGTTGAGTATTCAGAA |
